# The Analysis of *Pendolino* (*peo*) Mutants Reveals Differences in the Fusigenic Potential among *Drosophila* Telomeres

**DOI:** 10.1371/journal.pgen.1005260

**Published:** 2015-06-25

**Authors:** Giovanni Cenci, Laura Ciapponi, Marta Marzullo, Grazia D. Raffa, Patrizia Morciano, Domenico Raimondo, Romina Burla, Isabella Saggio, Maurizio Gatti

**Affiliations:** 1 Dipartimento di Biologia e Biotecnologie, Sapienza—Università di Roma, Roma, Italy; 2 Istituto Pasteur Fondazione Cenci Bolognetti, Sapienza—Università di Roma, Roma, Italy; 3 Dipartimento di Fisica, Sapienza—Università di Roma, Roma, Italy; 4 IBPM CNR, Sapienza—Università di Roma, Roma, Italy; NIH, UNITED STATES

## Abstract

*Drosophila* telomeres are sequence-independent structures that are maintained by transposition to chromosome ends of three specialized retroelements (HeT-A, TART and TAHRE; collectively designated as HTT) rather than telomerase activity. Fly telomeres are protected by the terminin complex (HOAP-HipHop-Moi-Ver) that localizes and functions exclusively at telomeres and by non-terminin proteins that do not serve telomere-specific functions. Although all *Drosophila* telomeres terminate with HTT arrays and are capped by terminin, they differ in the type of subtelomeric chromatin; the Y, XR, and 4L HTT are juxtaposed to constitutive heterochromatin, while the XL, 2L, 2R, 3L and 3R HTT are linked to the TAS repetitive sequences; the 4R HTT is associated with a chromatin that has features common to both euchromatin and heterochromatin. Here we show that mutations in *pendolino* (*peo*) cause telomeric fusions (TFs). The analysis of several *peo* mutant combinations showed that these TFs preferentially involve the Y, XR and 4th chromosome telomeres, a TF pattern never observed in the other 10 telomere-capping mutants so far characterized. *peo* encodes a non-terminin protein homologous to the E2 variant ubiquitin-conjugating enzymes. The Peo protein directly interacts with the terminin components, but *peo* mutations do not affect telomeric localization of HOAP, Moi, Ver and HP1a, suggesting that the *peo*-dependent telomere fusion phenotype is not due to loss of terminin from chromosome ends. *peo* mutants are also defective in DNA replication and PCNA recruitment. However, our results suggest that general defects in DNA replication are unable to induce TFs in *Drosophila* cells. We thus hypothesize that DNA replication in Peo-depleted cells results in specific fusigenic lesions concentrated in heterochromatin-associated telomeres. Alternatively, it is possible that Peo plays a dual function being independently required for DNA replication and telomere capping.

## Author Summary

Telomeres are specialized structures that protect chromosome ends from incomplete replication, degradation and end-to-end fusion. Abnormalities in telomere structure or maintenance can promote a variety of human diseases including premature aging and cancer. Although all human telomeres contain the same DNA sequences, they differ from each other in the subtelomeric regions or subtelomeres. Recent work has shown that human subtelomeres control telomere replication and that abnormalities in these structures can lead to localized chromosome instability and disease. However, the relationships between subtelomeres and telomeres are currently poorly understood. Here, we have addressed this problem using the fruit fly *Drosophila melanogaster* as model system. *Drosophila* subtelomers are very different from each other as they contain different types of chromatin. We have found that mutations in a gene we called *pendolino* (*peo*) cause telomeric fusions (TFs) and that these fusions preferentially involve the telomeres associated with a tightly packed form of chromatin called heterochromatin. Interestingly, none of the 10 mutants with TFs so far described in *Drosophila* shows the pattern of TFs observed in *peo* mutants. Thus, our data provide the first demonstration that subtelomeres can affect telomere fusion. We believe that these results will stimulate further studies on the role of subtelomeres in the maintenance of genome stability.

## Introduction

Telomeres are nucleoprotein complexes that counterbalance incomplete replication of terminal DNA and protect chromosome ends preventing both activation of cell cycle checkpoints and fusion events. In most organisms, the end replication problem is solved by telomerase, which mediates the addition of short GC-rich repeats to chromosome ends. These repeats specifically bind a discrete number of proteins, which recruit a series of additional factors to form large protein assemblies that ensure proper telomere function and homeostasis (reviewed in [1–4]). *Drosophila* telomeres are not elongated by telomerase but by targeted transposition of three specialized retroelements called *HeT-A*, *TART* and *TAHRE* (collectively abbreviated with HTT). However, *Drosophila* telomere formation does not require the HTT arrays; abundant evidence indicates that fly telomeres are epigenetically-determined structures that can assemble at the ends of the chromosomes independently of their terminal DNA sequence (reviewed in [5–7]).

With the exception of budding yeast, in organisms with telomerase telomeres are protected by the conserved shelterin complex. Human shelterin is a six-protein complex (TRF1, TRF2, POT1, TIN2, TPP1 and Rap1) that specifically associates with the telomeric TTAGGG repeats. TRF1, TRF2 directly bind the TTAGGG duplex and POT1 the single stranded overhang. TIN2 and TPP1 do not bind DNA and interconnect TRF1 and TRF2 with POT1. TRF2 interacts with hRap1, a distant homologue of *S*. *cerevisiae* Rap1. The shelterin subunits share properties that distinguish them from the non-shelterin telomere-associated proteins: they are specifically enriched at telomeres throughout the cell cycle and appear to function only at telomeres [1]. The human non-shelterin proteins, which are not telomere-specific in localization and function, include the conserved CST complex, HP1a, and proteins involved in DNA repair and/or replication such as the ATM kinase, the Ku70/80 heterodimer, the MRE11/RAD50/NBS1 (MRN) complex, Rad51, the ERCC1/XPF endonuclease, the Apollo exonuclease, the FEN1 nuclease, the RecQ family members WRN and BLM; the RTL1 helicase, RPA70, the Timeless component of the replisome, and the subunits of the conserved ORC prereplication complex. Depletion of any one of the shelterin subunits or the shelterin-associated proteins leads to visible telomere defects ranging from altered packaging of telomeric chromatin (multi telomere signals after FISH), telomere loss and telomere fusion (reviewed in [1–4]; see also [8, 9]).

Most of the *Drosophila* telomere-capping proteins have been identified by molecular cloning of genes specified by mutations that cause telomeric fusions (TFs) in larval brain cells. Genetic and molecular analyses have thus far identified 11 loci that are required to prevent TF (henceforth they will be designated as TF genes). These are *effete* (*eff*; also called *UbcD1*) that encodes a highly conserved E2 enzyme that mediates protein ubiquitination [10, 11], *Su(var)205* that encodes Heterochromatin Protein 1a (HP1a) [12], the *Drosophila* homologues of the *ATM*, *RAD50*, *MRE11* and *NBS1* DNA repair genes [13–19], *without children (woc)* that specifies a transcription factor [20]; *caravaggio* (*cav*), *modigliani (moi)*, *verrocchio (ver)* and *hiphop* that encode the components of the terminin complex [21–25]. HOAP (the *cav* product HP1/ORC-Associated Protein [21–26]), Moi and HipHop are fast evolving non-conserved proteins that do not share homology with any known telomere-associated protein [22–24]Ver is also a fast evolving protein; however, it contains an OB-fold motif that is structurally homologous to the OB fold of the Stn1 protein of the conserved CST complex [23]. Although the structural characterization of terminin is still incomplete, the extant data suggest that HOAP and HipHop are primarily bound to the DNA duplex while Ver is associated with the single-stranded overhang [6, 22, 23, 26]. In contrast with the other telomere capping proteins (Eff, HP1a, ATM, Mre11, Rad50, Nbs, and Woc) that have multiple localizations and functions, HOAP, HipHop, Moi and Ver localize only at telomeres and appear to function only in telomere maintenance. These properties are similar to the shelterin properties, suggesting that terminin is a functional analog of shelterin [25]. Furthermore, the findings that shelterin subunits are not conserved in flies, that terminin components have no homologues (with the possible exception of Ver) outside Drosophilidae, and that terminin subunits are encoded by fast evolving genes have suggested a hypothesis on terminin evolution. We proposed that the transition between a telomerase-driven and a transposon-driven telomere elongation mechanism generated a divergence in terminal DNA sequences, which exerted a strong selective pressure towards the evolution of sequence-independent telomere binding proteins such as those that comprise terminin. We also hypothesized that non-terminin *Drosophila* telomere-capping proteins with multiple localizations and functions correspond to ancestral telomere components that did not evolve as rapidly as terminin because of the functional constraints imposed by their participation in diverse cellular processes [23–25].

In addition to their peculiar telomere elongation mechanism, *Drosophila* telomeres are also characterized by striking variations in their subtelomeric regions. Recent work has shown that subtelomeric regions play important regulatory roles in mammalian telomere behavior. For example, it has been reported that most human telomeres are replicated by forks progressing from subtelomere to telomere [27] and that the timing of telomere replication depends on the type of subtelomeric DNA; the telomeres associated with satellite-like subtelomeric sequences replicate later than telomeres that are not associated with this type of subtelomeric DNA [28]. Furthermore, recent work has shown that chimpanzee telomeres carrying subtelomeric heterochromatin replicate later than telomeres devoid of heterochromatic subtelomeres [29]. However, the fusigenic properties of mammalian telomeres carrying different subtelomeres have never been investigated.

*Drosophila* is an ideal model organism for investigating the influence of subtelomeric regions on telomere behavior. All *Drosophila* telomeres terminate with HTT arrays that are capped by terminin; these HTT arrays are juxtaposed to different types of chromatin: canonical constitutive heterochromatin (the Y, XR, and 4L telomeres), clusters of repetitive telomere-associated sequences (designated as TAS, and present at the XL, 2L, 2R, 3L and 3R telomeres), or sequences with both euchromatic and heterochromatic features (4R telomeres). Here we describe a *Drosophila* gene, *pendolino* (*peo*), identified by mutations that preferentially induce TFs between telomeres associated with constitutive heterochromatin. The Peo protein binds terminin but does not have the typical terminin properties, as it is conserved in mammals and associates with several chromosomal sites. In addition, Peo is required for PCNA recruitment and for general DNA replication. However, both the present and previous results strongly suggest that telomere lesions generated by general defects in DNA replication are unable to induce TFs in *Drosophila* cells. We thus propose that loss of *peo* function results in specific fusigenic lesions concentrated in heterochromatin-associated telomeres, and that these lesions might be generated during telomere replication.

## Results

### Isolation and characterization of *pendolino (peo)*

**Fig 1:**
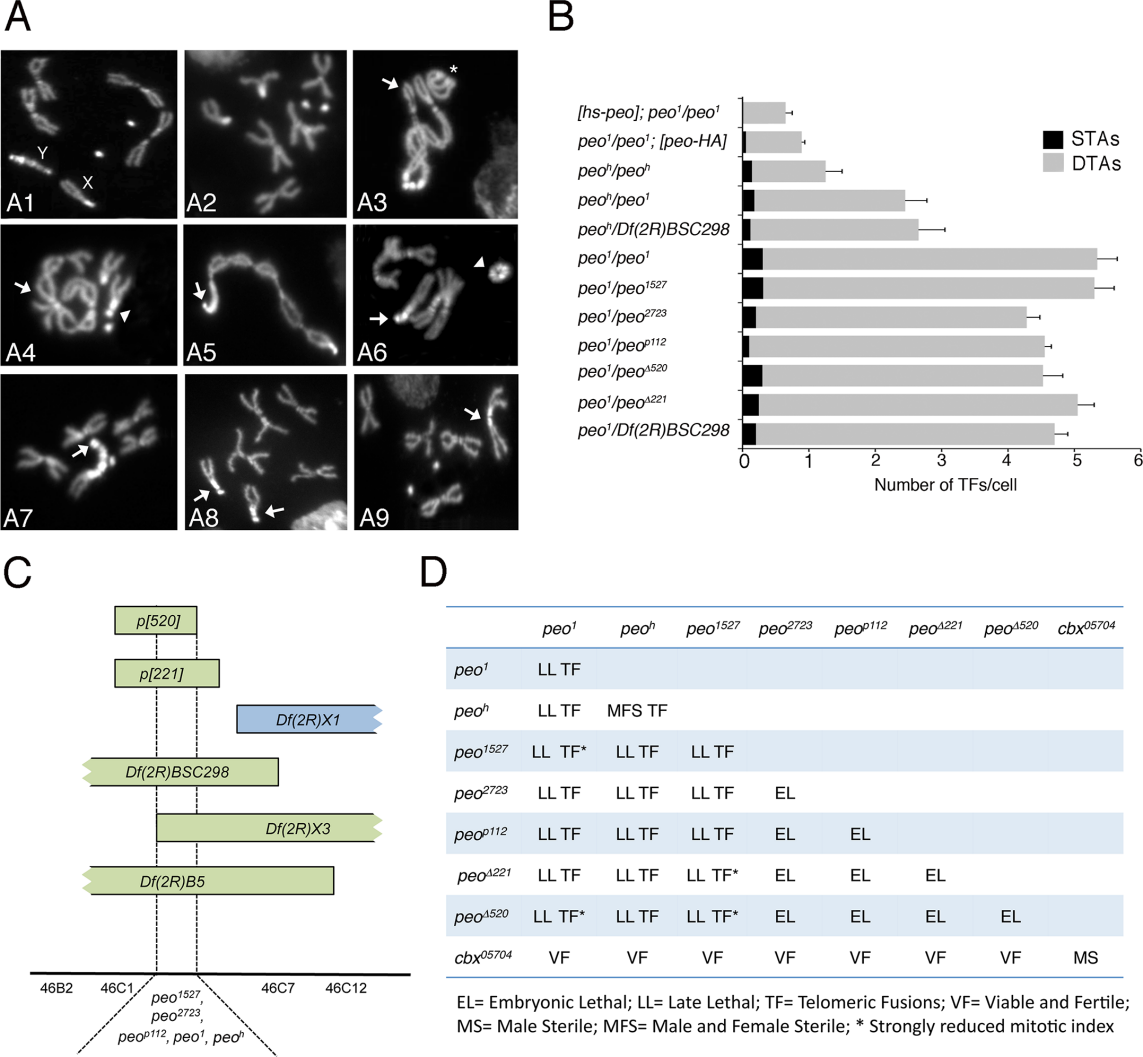
Mutations in *peo* cause telomeric fusions. (A) Examples of TFs in *peo* mutant neuroblasts. (A1-2) Control (*Oregon-R*) male (A1) and female (A2) metaphases; (A3-A5) *peo* ^*1*^ */peo* ^*1*^ metaphases showing: (A3) a ring autosome (abbreviated with A; asterisk) and, starting from the arrow, a multicentric chromosome A-XL∙XR-4-4-XR∙XL-A-A; (A4) an XR-4 TF (arrowhead) and an A-A-A-A multicentric chromosome (arrow); (A5) a 4 (arrow)-XR∙XL-A-A-A-A-XL∙XR-4 multicentric chromosome including the entire female complement. (A6-A9) *peo* ^*h*^ */peo* ^*h*^ metaphases showing: (A6) a ring Y chromosome (arrowhead) and a 4 (arrow)-4-XR tricentric chromosome; (A7) a 4 (arrow)-YS∙YL-XR tricentric chromosome; (A8) two 4-XR TFs (arrows); (A9) an XR-XR TF (arrow). (B) Frequencies of TFs in *peo* mutant combinations. *hs-peo* and *peo-HA* are rescued by a construct carrying a wild type copy of the *peo* gene. At least 250 cells from at least 4 brains were scored for each genotype. (C) Deficiency mapping of *peo*. (D) Complementation analysis showing the phenotypes of animals heterozygous for the indicated genes/alleles.

The *pendolino* ^*1*^ *(peo* ^*1*^ *)* mutation was isolated by a cytological screen of 120 late lethal mutants mapping to the second chromosome, recovered after I element mobilization by I-R dysgenic crosses (see Materials and Methods). Mitotic cells of DAPI-stained brain preparations from *peo* ^*1*^ */peo* ^*1*^ larvae displayed very frequent telomeric fusions (TFs; Fig 1A and 1B), often resulting in multicentric linear chromosomes that resemble little “trains” of chromosomes. The *pendolino* gene was named after this phenotype just as *caravaggio*, *modigliani* and *verrocchio*, which are all names of Italian trains.

Recombination analysis with visible markers and deficiency mapping placed *peo* ^*1*^ in the 46B-C polytene chromosome interval uncovered by *Df(2R)X3* and *Df(2R)B5* but not by *Df(2R)X1* (Fig 1C). Previous studies mapped to this interval 3 lethal complementation groups [30]. *peo* ^*1*^ failed to complement the *1527* and *2723* mutant alleles (henceforth designated as *peo* ^*1527*^ and *peo* ^*2723*^) of group III for both the lethality and the TF phenotype but complemented representative alleles of groups I and II (Fig 1D). *peo* ^*1*^ also failed to complement the P element insertion *p112* (henceforth *peo* ^*p112*^) and the small *p221* and *p520* deficiencies (henceforth *peo* ^*∆221*^ and *peo* ^*∆520*^) all generated by the remobilization of the *P{w* ^*+*^, *ry* ^*+*^ *}AJN2* insertion [31], originally localized proximally to the *Df(2R)X3* breakpoint (Fig 1C and 1D). Finally, we identified another *peo* mutant allele (*peo* ^*h*^) by a cytological screen of a collection of 193 late lethal mutants that arose in the Zucker’s collection of heavily mutagenized viable lines (see Materials and Methods for details). *peo* ^*h*^ is homozygous viable but male and female sterile, and it is lethal in combination with *peo* ^*1*^ (*peo* ^*1*^/*peo* ^*h*^); the lethal phases of selected combinations of *peo* mutant alleles and deficiencies are reported in Fig 1D.

**Fig 2:**
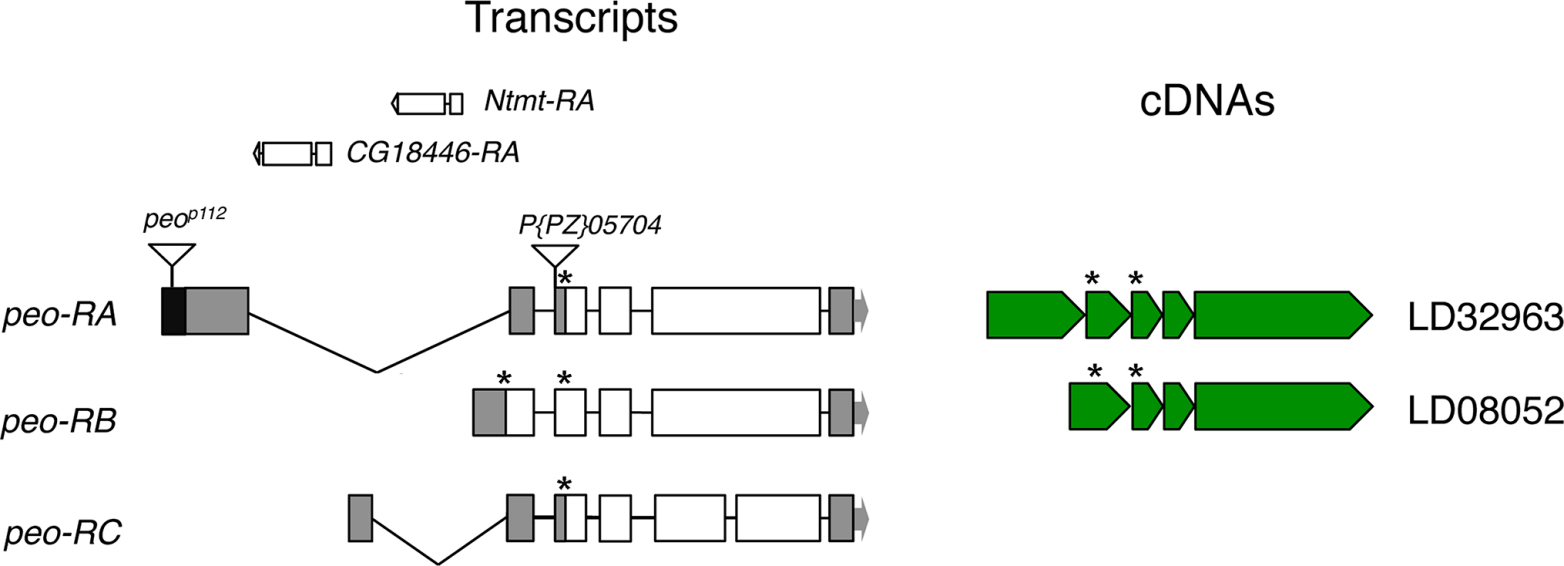
Structure of the *peo* (*CG10536)* transcripts and available cDNAs. The triangles indicate P element insertions; *P[PZ]05704* is not responsible for the *crossbronx (cbx)* phenotype (see text for detailed explanation). The *Ntmt* and *CG18446* genes are nested into the introns defined by the *peo*-RA and *peo*-RC transcripts. The asterisks indicate the positions of ATG start codons.

To identify the *peo* gene at the molecular level we exploited the *peo* ^*p112*^ allele that carries a *P*{*w* ^*+*^, *ry* ^*+*^}construct inserted into the gene [30]. Using inverse PCR we found that the P construct is inserted into the 5’ UTR of the longest transcript of the *CG10536* gene (Fig 2). *CG10536* was originally named *ms(2)46C* [32] an then renamed *crossbronx (cbx*) [33]. However, *CG10536* does not correspond to *ms(2)46C*/*cbx*. The phenotype associated with the *ms(2)46C*/*cbx* mutation was attributed to the *P{PZ}05704* insertion that maps just proximal to the UTR region of *CG10536* (Fig 2). However, this attribution was only tentative because the male sterile phenotype was neither mapped over deficiency nor reverted by P element excision [32]. We found that males homozygous for the *P{PZ}05704* insertion are sterile and show the spermatid abnormalities previously described [32]. In contrast, males bearing the same insertion over *Df(2R)B5* that uncover the 46B-C interval were fully fertile and displayed normal spermatids (Fig 1D). We thus conclude that the *ms(2)46C*/*cbx* mutation maps outside the 46B-C region, and that *CG10536* actually corresponds to *pendolino*.

The organization of the *peo* locus is rather complex. The gene encodes three different transcripts; the long introns of two of these transcripts contain genes (*Ntmt* and *CG18446*) with an opposite transcriptional orientation to *peo/CG10536* (FlyBase; Fig 2). The three putative *peo* transcripts encode proteins of 183, 214 and 244 aa and are identified by 2 cDNAs (FlyBase, Fig 2). These proteins share homology with the E2 variant ubiquitin-conjugating enzymes, which are devoid of the catalytic cysteine that mediates ubiquitin transfer [34]. Interestingly, *peo* has an intronless paralogue (*CG16894*) that maps to the 56F9 polytene chromosome region. The CG16894 protein shares 35.4 identity and 51.6 similarity with Peo and its function is currently unknown (FlyBase).

Sequencing of *peo* ^*1*^ did not revealed nonsense, frameshift, splice-site or missense mutations in the protein-coding sequences of *CG10536* with respect to the FlyBase sequences. In addition, sequencing of approximately 2000 bp upstream of the gene ATG and in situ hybridization experiments did not reveal I element insertions. Nonsense, frameshift or splice-site mutations were also absent from the protein coding sequences of *peo* ^*1527*^ and *peo* ^*h*^ mutant genes. All *peo* mutant alleles displayed several genomic single nucleotide polymorphisms (SNPs) with respect to the Fly Base sequence. However these SNPs were all present in DPGP natural populations (www.dpgp.org/dpgp3), suggesting that they are associated with little if any phenotypic consequences. Collectively, these results suggest that *peo* ^*1*^, *peo* ^*1527*^ and *peo* ^*h*^ are regulatory mutations that lower the intracellular amount of the Peo protein (see below). However the molecular lesions in these mutant alleles remain to be identified.

To unambiguously determine the identity of *peo* we performed rescue experiments using the LD08052 cDNA clone (Fig 2; see also FlyBase). Sequencing confirmed that this cDNA contains the entire coding sequence of the *CG10536-RB* transcript, which produces the larger protein isoform encoded by the locus (Fig 2). The LD08052 cDNA was fused in frame with the heat shock (*hsp70*) promoter or used to make a construct containing the tubulin promoter and a 3HA sequence at the 3’ of the gene. Both constructs were then used to make transgenic flies and both rescued the lethality and the TF phenotype of the *peo* ^*1*^ mutant flies. In *hs-CG10536; peo* ^*1*^ */peo* ^*1*^ flies exposed to heat shocks (1h at 37°C every 24 h throughout development), survival of *peo* ^*1*^ */peo* ^*1*^ individuals with respect their *peo* ^*1*^ */CyO* siblings was 18% of the Mendelian expectation, and the TF frequency dropped to less than 1 per cell from more than 5 per cell (Fig 1B). In the presence of the *Tub-CG10536-3HA* construct, the survival rate of *peo* ^*1*^ */peo* ^*1*^ homozygotes with respect to *peo* ^*1*^ */CyO* heterozygotes was 10% and the TF frequency less than 1/cell (Fig 1B). To confirm these rescue data we generated *peo* ^*h*^ */peo* ^*h*^ larvae bearing the *Tub-CG10536-3HA* construct. The brains of these larvae displayed a 5-fold reduction in TF frequency compared to those of *peo* ^*h*^ */peo* ^*h*^ larvae (0.2 *vs* 1 TF/ cell). Thus, our results collectively indicate that *CG10536* corresponds to *peo*.

### Peo specifically affects heterochromatin-associated telomeres

In most colchicine-treated metaphases from *peo* ^*1*^ */peo* ^*1*^, *peo* ^*1*^ */ peo* ^*1527*^, *peo* ^*1*^ */peo* ^*2723*^, *peo* ^*1*^ */peo* ^*p112*^, *peo* ^*1*^ */ peo* ^*∆221*^, *peo* ^*1*^ */peo* ^*∆520*^ and *peo* ^*1*^ */Df(2R)BSC298* (henceforth *Df(2R)BSC298* will be abbreviated with *Df)*, the majority of telomeres were involved in fusions, often forming tangles of chromosomes difficult to resolve (Fig 1A and 1B). However, a careful examination of these tangles led us to estimate the average number of TFs per cell and to determine the relative frequencies of single telomere associations (STAs) and double telomere associations (DTAs). STAs involve a single telomere that fuses with either its sister telomere or another single nonsister telomere. In DTAs, two sister telomeres fuse with another pair of sister telomeres. STAs are likely to be generated during the S-G2 phase, while DTAs are thought to result from the replication of TFs generated during G1 [10, 35]. In *peo* mutants, DTAs were much more frequent than STAs (Fig 1B) just as in the other TF mutants in which the relative frequencies of STAs and DTAs have been determined, namely *eff*, *Su(var)205*, *cav*, *mre11*, *rad50*, *nbs*, *tefu (ATM)*, *woc*, *moi* and *ver* [10, 12, 15, 16, 21, 20, 23, 24]. This bias towards DTAs may reflect the proximity of *Drosophila* telomeres during G1 that would be progressively lost as cells proceed through S and G2 [10, 36]. It has been also suggested that telomere fusion occurs primarily during G1, because the DNA repair pathways that join chromosome ends are more active in G1 than in S or G2 [37].

**Fig 3:**
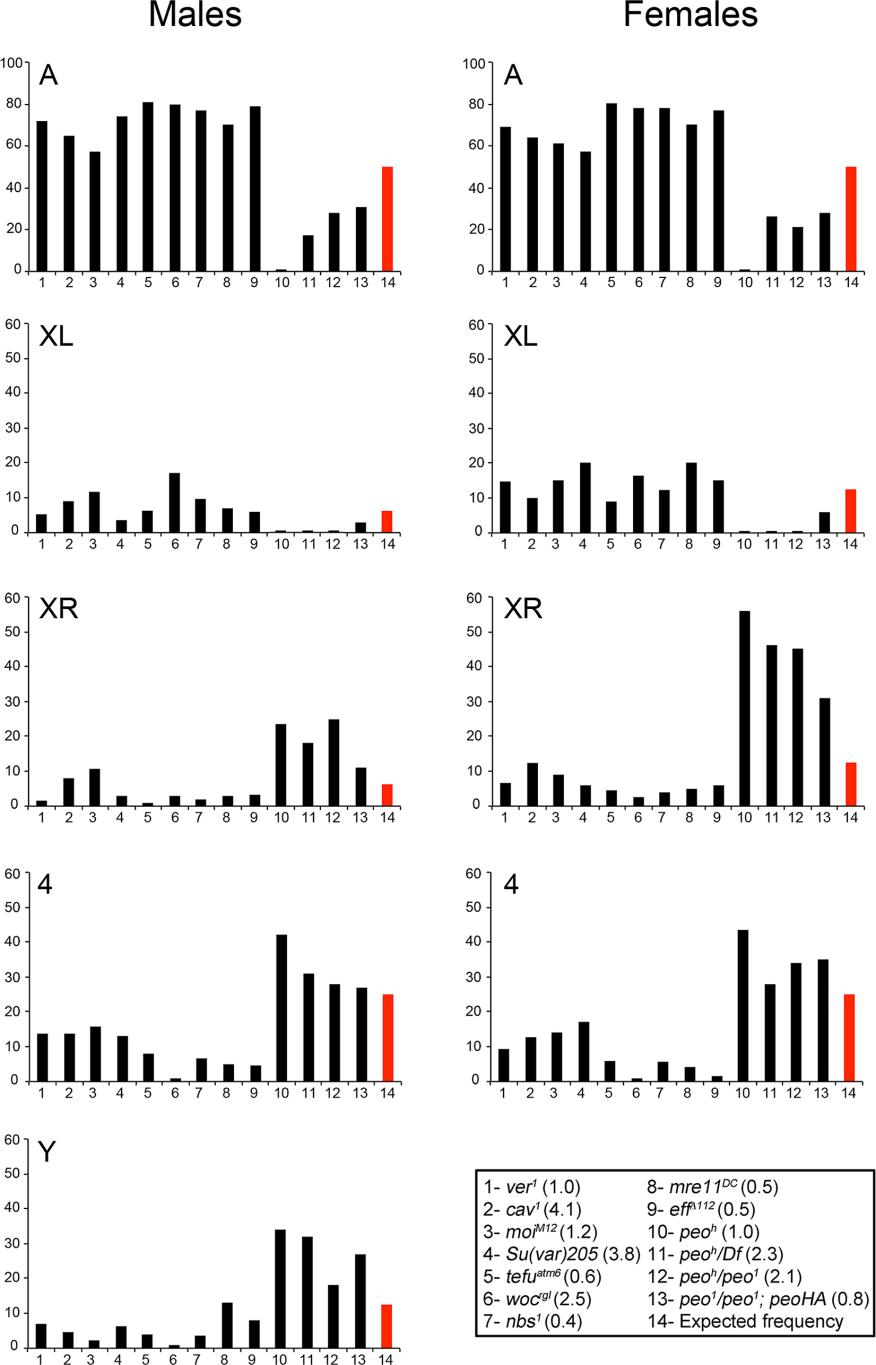
Involvement of individual telomeres in fusion events in different TF mutants. The vertical axes of the graphs show the frequencies (%) with which individual telomeres are involved in TFs. The red columns indicate the expected frequencies assuming a random involvement of telomeres in fusion events. The columns in the graph correspond to numbered genotypes in the box, and the numbers in parentheses next to each genotype indicates the observed TF frequencies. At least 122 fused telomeres from at least 4 brains were scored for each genotype. The *Su(var)205* mutant (# 4) is a *Su(var)205* ^*04*^ */Su(var)205* ^*05*^ heteroallelic combination. The observed number of "heterochromatic telomeres" involved in TFs in each *peo* mutant combination is significantly higher than would be expected by chance (p < 0.001 in Chi-square test). In contrast, in all other mutants, this number is significantly lower than the expected one (p < 0.001 in Chi-square test). All observed and expected values and the statistical analysis are presented in detail in S1 Table.

Although *peo* mutants show a high DTA/STA ratio as the other TF mutants, they exhibit a specific pattern of TFs. The analysis of the *peo* ^*h*^ mutant that shows ~1 TF/cell allowed a very precise definition of the telomeres involved in fusion events. In *peo* ^*h*^ homozygous brains of males and females, nearly all TFs involved the telomeres associated with the heterochomatic regions of the chromosomes, namely those of the entirely heterochromatic Y chromosome (YS and YL), the telomere of the right arm of the X chromosome (XR) and the fourth chromosome telomeres (Fig 1A). Of the two telomeres of the fourth chromosome only one is associated with constitutive heterochromatin (4L) but we were not able to distinguish between 4L and 4R, as the DAPI-stained fourth chromosomes appear as brightly fluorescent dots in which the chromosome arms are not discernible. High frequencies of TFs between heterochromatin-associated telomeres (henceforth abbreviated with Ha-telomeres) were also observed in *peo* ^*h*^ */Df* and *peo* ^*h*^ */peo* ^*1*^ brains (Fig 3). We note that we classified as DTAs all TFs between Ha-telomeres, because the close apposition of the sister chromatids in heterochromatin does not allow a distinction between STAs and DTAs. Thus, the apparent lack of STAs observed in weak *peo* mutants (Fig 1B) might not reflect a real absence of this type of TFs.

The high incidence of TFs between Ha-telomeres is not due to an allele-specific effect of *peo* ^*h*^, as a high frequency of TFs involving the Ha-telomeres was also observed in *peo* ^*1*^ */peo* ^*1*^ mutant cells bearing the *Tub*-*peo* ^*+*^ *-3HA* rescue construct (Fig 3). This suggests that the Ha-telomeres are preferentially affected by an impairment of *peo* function and that this effect is partially masked in strong mutants in which most telomeres are fused. The TF pattern observed in *peo* mutants is highly specific, as none of the TF mutants we characterized in the past showed a prevalence of TFs between the Ha-telomeres. To precisely compare the patterns of fusions we re-examined all extant *Drosophila* TF mutants (*eff*, *cav*, *Su(var)205*, *mre11*, *rad50*, *nbs*, *tefu*, *woc*, *moi* and *ver*). All these mutants displayed an inverse TF pattern compared with *peo* mutants, with lower than expected frequencies of fusions involving the Y, the XR, and the 4^th^ chromosome telomeres and higher than expected frequencies of TFs between the telomeres of the major autosomes (Fig 3 and S1 Table). We also confirmed that *eff* mutants do not exhibit a prevalence of fusion between Ha-telomeres (Fig 3) [10]. This finding strongly suggests that the canonical E2 ubiquitin conjugating enzyme encoded by *eff* and the E2 variant encoded by *peo* do not function in the same telomere protection pathway.

**Fig 4:**
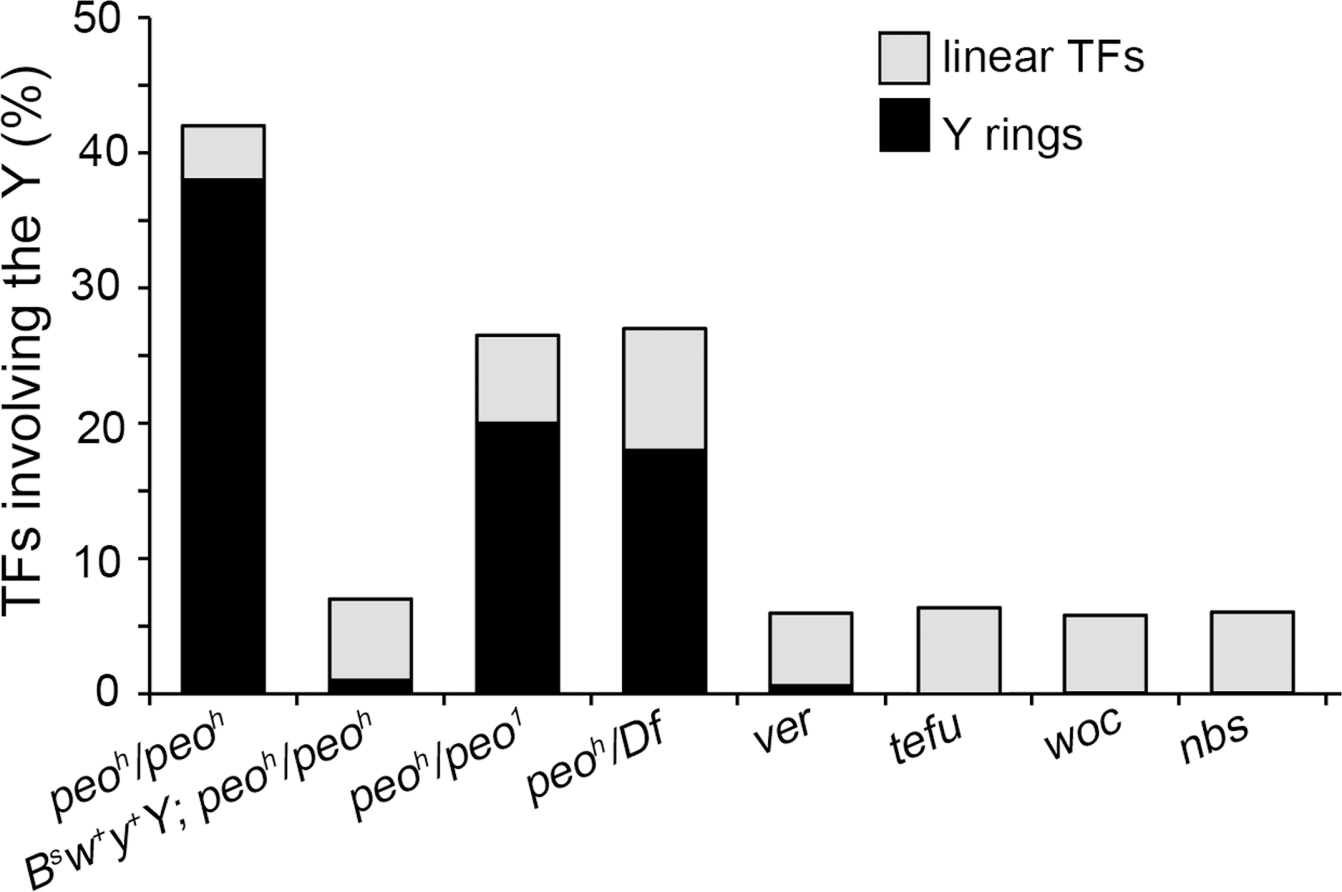
Y chromosome ring formation in *peo* mutants. *peo* mutants brains exhibit an extremely high Y-ring frequency that is not observed in other TF mutants. The frequency of Y rings in *peo* mutants is much higher than expected assuming a random involvement in TFs of the heterochromatin-associated telomeres (see text) and is strongly reduced when mutants bear a *B* ^*S*^ *w* ^+^ *y* ^+^Y that carries a segment of euchromatin appended to the end of its left arm.

In interphase nuclei, the heterochromatic regions of the chromosomes aggregate to form an irregular mass of chromatin called chromocenter [38]. Thus, the specific pattern of TFs observed in *peo* mutants could depend either on an abnormal organization and compaction of the chromocenter or on a specific chromatin composition of heterochromatic telomeres. To discriminate between these possibilities we introduced in a *peo* ^*h*^ mutant background a *B* ^*s*^ *w* ^*+*^ *y* ^*+*^ Y chromosome that carries a euchromatic fragment marked with *B* ^*s*^ *w* ^*+*^ and *y* ^*+*^ at its YL end [39] (see also FlyBase). We found that in peo^*h*^ mutant brains the frequency with which the *B* ^*s*^ *w* ^*+*^ *y* ^*+*^ Y chromosome is involved in TFs is more than 5-fold lower than that of a normal Y (Fig 4). Specifically, the formation of Y rings, which in *peo* ^*h*^ */peo* ^*h*^ mutants represents ~90% of the total fusions involving the Y (that together are more than 40% of the observed TFs), was drastically reduced in the presence of a *B* ^*s*^ *w* ^*+*^ *y* ^*+*^ Y. Because the cells bearing a *B* ^*s*^ *w* ^*+*^ *y* ^*+*^ Y chromosome do not exhibit detectable morphological variation in the chromocenter compared to wild type, these results strongly suggest that the preferential involvement of the Ha-telomeres in *peo*-induced TFs is a consequence of their association with heterochromatin and not of their arrangement within the interphase nucleus.

The particularly high frequency of Y rings in *peo* mutants is another peculiar feature of their TF pattern. Indeed, the Y rings are rare or virtually absent in mutants in genes such as *ver*, *tefu*, *woc*, and *nbs* (Fig 4). On the assumption of a random involvement of telomeres in TFs, the expected frequency of Y ring is 1/15 of the total fusions involving the Y chromosome and 1/6 of the fusions involving the Y and either the XR, or the 4^th^ chromosome telomeres. Thus, formation of Y rings in *peo* mutants is specifically and strikingly frequent. This could reflect a particularly high frequency of fusigenic lesions on the Y telomeres, or the contemporary presence of these lesions on the opposite Y telomeres, or both.

### Peo interacts with terminin

We have recently observed that brains from larvae heterozygous for both *Su(var)205* ^*05*^ and *cav* exhibit ~ 0.1 TFs/cells, while in wild type, *Su(var)205* ^*05*^ */+*, *cav/+* brains the TF frequency was virtually zero (300 metaphases analyzed in each case). This finding suggests that *Su(var)205* and *cav* genetically interact and that a simultaneous reduction of HP1a and HOAP results in a low level of TFs. We thus asked whether *peo* exhibits similar genetic interactions with other TF mutants. Double heterozygotes for *peo* ^*1*^ and either *cav* ^*1*^, *moi* ^*1*^, *ver* ^*2*^, *Su(var)205* ^*04*^ or *Su(var)205* ^*05*^ displayed TF frequencies ranging from 0.05 to 0.1 per cell, whereas heterozygotes for *peo* ^*1*^ and either *eff* ^*Δ73*^, *woc* ^*B111*^, *mre11* ^*DC*^, *rad50* ^*Δ5*.*1*^, *nbs* ^*1*^ or *tefu* ^*atm6*^ did not exhibit TFs (in all cases, we examined at least 300 metaphases).

**Fig 5:**
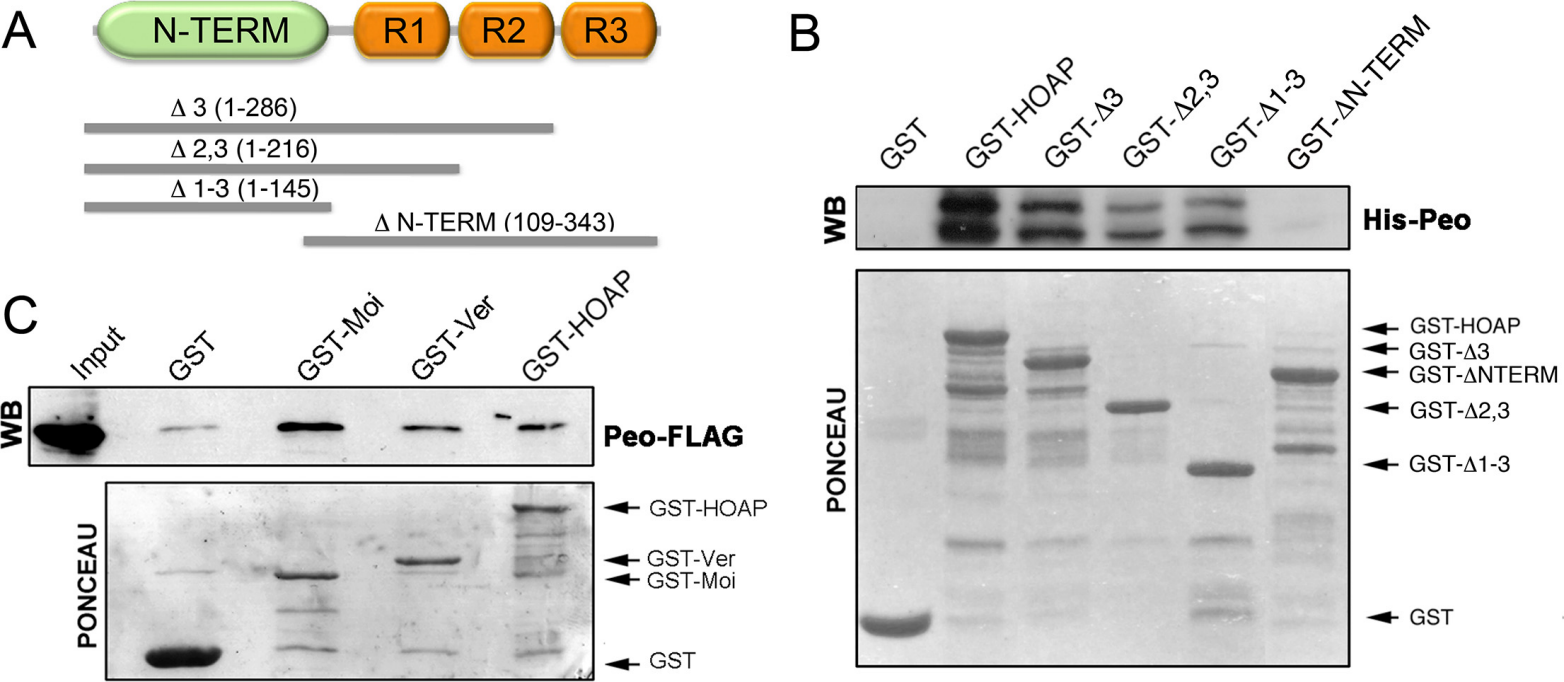
Peo directly interacts with HOAP and is also likely to bind Moi and Ver. (A) Schematic of HOAP truncations used in GST pulldown experiments; R indicates the repeated segments found in the C-proximal half of the protein. (B) Bacterially purified GST-HOAP segments spanning the N proximal half of the protein precipitate bacterially expressed His-Peo, which is not pulled down by GST alone or the C-proximal half of HOAP. His-Peo was detected using our anti-Peo antibody; the two Peo bands are likely to correspond to different translation products generated in *E*. *coli* starting from the two closely spaced ATG codons present on *peo* cDNA (see Fig 2) (C) GST-HOAP, GST-Moi and GST-Ver precipitate Peo-FLAG from HeLa cell extracts. Peo-FLAG was detected with anti-FLAG antibody.

We next asked whether the Peo protein physically interacts with the terminin components. A preliminary experiment using the yeast two-hybrid assay suggested that Peo directly interacts with HOAP but not with HP1a (S1 Fig). To confirm this result we performed a GST pulldown assay using bacterially expressed 6His-Peo and GST-tagged HOAP polypeptides of different length. As shown in Fig 5A and 5B, the intact HOAP protein and the HOAP fragments containing the N-terminal region of the protein (aa 1–145) precipitated Peo, while a larger HOAP fragments including the 3 repeated segments of the protein (aa 109–343) failed to bind Peo. We then investigated whether Peo interacts with Moi and Ver. We performed GST pulldown experiments with extracts from human 293T cells expressing Peo-FLAG and either GST alone, GST-Moi, GST-Ver or GST-HOAP. We chose to express *Drosophila* tagged proteins in human cells because a heterogeneous cellular environment is likely to reduce the probability of indirect interactions among fly proteins. As shown in Fig 5C, Peo-FLAG is precipitated by GST-Moi, GST-Ver and GST HOAP but not GST alone. Collectively, these results provide strong evidence that Peo directly binds HOAP; in addition, they suggest that Peo directly interacts with Moi and Ver.

### Mapping the Peo sites that interact with the terminin proteins

**Fig 6:**
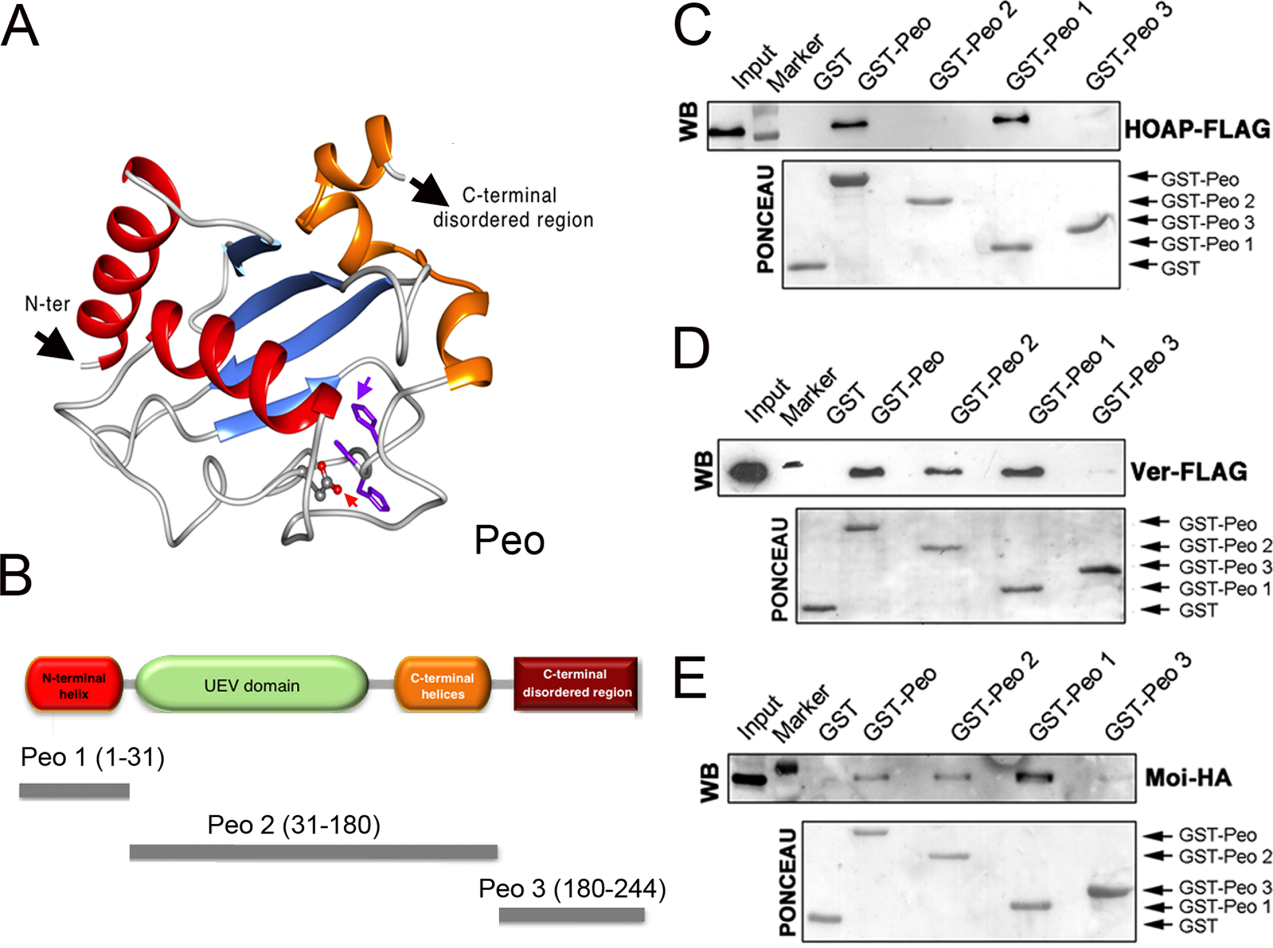
Mapping the Peo regions that interact with terminin. (A) A tridimensional molecular model for Peo. The arrow pointing to “N-ter” indicates the N-terminus of the protein; the arrow pointing to “C-ter” indicates the starting site of the disordered C-terminal region of Peo (not depicted); the variant Asp residue and His-Pro-His motif are represented as sticks and indicated by red and purple arrows, respectively (see Materials and Methods and S2 Fig for construction of the Peo 3D model). (B) Schematic organization of the Peo protein and Peo truncations used for GST pulldown. (C-E) GST-pulldown from S2 cells extracts expressing HOAP-FLAG (C), Ver-FLAG (D) or Moi-HA (E). HOAP-FLAG, Ver-FLAG and Moi-HA were detected with anti-FLAG and anti-HA antibodies. The C-terminal disordered region (included in the Peo 3 fragment) does not interact with any of the terminin components. HOAP specifically interacts with N-terminal region of Peo; in contrast, Moi and Ver interact with both the N terminal and the UEV-containing central regions of the protein.

*peo* encodes an E2 variant (UEV) enzyme; the UEV proteins are similar to the E2 ubiquitin conjugating enzymes (UBCs) but lack the catalytic cysteine residue that mediates the interaction between ubiquitin and E2 [40]. We elaborated a three-dimensional model of Peo exploiting a series of bionformatic analyses (Fig 6A; see also Material and Methods and S2 Fig). We confirmed that Peo lacks the catalytic cysteine of E2 enzymes. In addition, 8 residues before the catalytic cysteine site, Peo exhibits an HPH tripeptide (S2 Fig) instead of HPN, which is a canonical signature of the E2 superfamily [41]. Peo contains a UEV domain of ~150 amino acids resembling the canonical E2 fold in its hydrophobic core and active site region. This domain consists of three helices packed against a four-stranded antiparallel β-sheet. Next to the UEV domain, Peo contains two C-terminal helices that are present in all E2 proteins but missing in other E2 variant enzymes such as Tsg101 and Mms2. Finally, prediction of potentially disordered regions revealed that Peo also contains a long (50 aa) disordered region at the C-terminus (Figs 6A and S2; see also Materials and Methods).

To map the Peo sites that interact with the terminin proteins, we subdivided Peo into 3 GST-tagged fragments: Peo 1 (aa 1–31) that contains the Peo N terminal region which is absent in the short isoform (see Fig 2); Peo 2 (aa 31–180) that includes the central UEV domain of the protein; and Peo 3 (aa180-244) that contains C-terminal disordered region of Peo (Fig 6B). These bacterially expressed GST-tagged Peo fragments were then used to probe *Drosophila* S2 cell extracts expressing HOAP-HA, Moi-HA or Ver-FLAG (Fig 6C–6E). GST pulldown showed that both Moi and Ver interact with the entire Peo protein (GST-Peo), GST-Peo 1 and GST-Peo 2 but not with GST-Peo 3 or GST alone (Fig 6D and 6E). HOAP did not display the same interaction pattern as Moi and Ver. It was precipitated by GST-Peo and GST-Peo 1 but not by GST-Peo 2 and GST-Peo 3 and thus failed to interact with the Peo UEV domain (Fig 6C).

### Peo is not required for terminin recruitment at telomeres

The finding that Peo binds HOAP, Moi and Ver prompted us to ask whether Peo is required for terminin localization at chromosome ends. Although Peo does not appear to interact with HP1a, we also asked whether loss of the wild type function of *peo* affects HP1a localization at telomeres. Of the latter proteins, only HOAP is clearly detectable at both mitotic and polytene chromosome telomeres; HP1a, Moi and Ver can be easily detected at polytene chromosome ends but not at mitotic telomeres [23, 24, 42]. We thus analyzed HOAP localization in both mitotic and polytene chromosomes of *peo* ^*1*^ homozygous mutants; HP1a, Moi and Ver localization was instead studied only in *peo* ^*1*^ polytene chromosomes.

**Fig 7:**
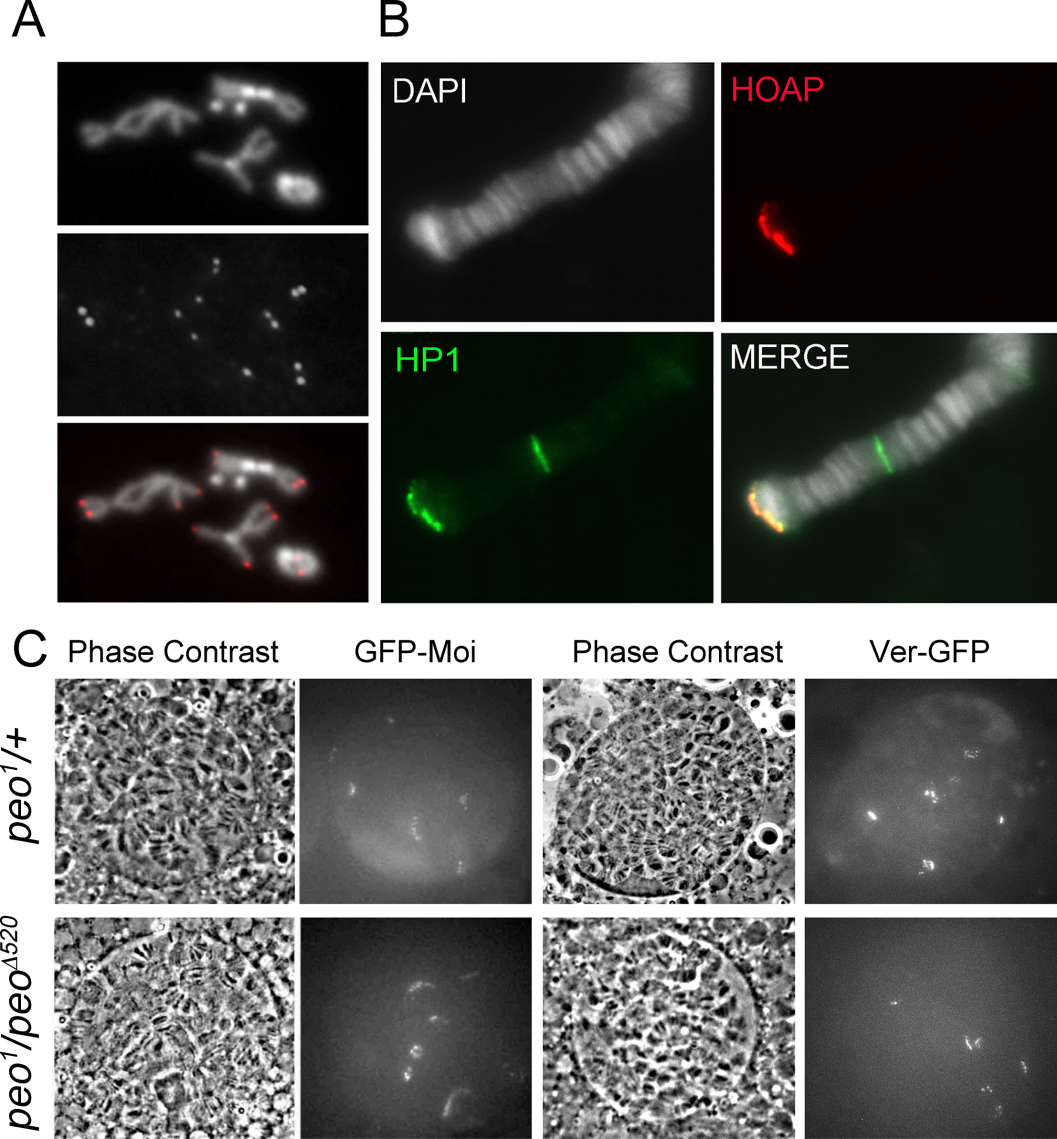
Terminin and HP1a localize normally at the telomeres of *peo* mutant cells. (A) HOAP is normally enriched at the telomeres of brain cell metaphases *peo* ^*1*^ */peo* ^*1*^ mutants (B). HOAP and HP1a localize normally to the telomere of *peo* ^*1*^ */peo* ^*1*^ polytene chromosomes (C) Normal localization of Ver-GFP and GFP-Moi in live, unsquashed salivary gland nuclei from *peo* ^*1*^ */peo* ^*∆520*^. Note that these nuclei display 6 discrete fluorescent signals that are likely to correspond to the telomeres of XL, 2L, 2R, 3L, 3R and 4R.

An analysis of mitotic chromosomes immunostained for HOAP revealed that mutations in *peo* do not substantially affect HOAP localization at telomeres, as the frequency of HOAP-stained telomeres in *peo* mutants and the intensity of the signals were similar to those observed in wild type controls (Fig 7A). Consistent with these results, immunostaining with anti-HOAP and ant-HP1a antibodies showed that *peo* ^*1*^ */Df* mutants exhibit normal concentrations of these proteins at polytene chromosome telomeres (Fig 7B). Because antibodies to Moi or Ver are not currently available, to analyze the localization of these proteins in *peo* mutants we constructed flies expressing GFP-tagged forms of Moi or Ver in a *peo* ^*1*^ mutant background. The analysis of unfixed polytene chromosome nuclei from *peo* ^*1*^ */peo* ^*1*^ flies expressing either GFP-Moi or Ver-GFP revealed that they exhibit 6 discrete GFP signals (Fig 7C), which we have previously shown to correspond to the 6 euchromatic telomeres of polytene chromosomes [23, 24]. In addition, we found that the intensities of these signals were comparable to those observed in *peo* ^*1*^ */+* heterozygotes (Fig 7C). Collectively, these results indicate that the wild type function of *peo* is not required for telomeric localization of HOAP, Moi, Ver and HP1a, and that the strong telomere fusion phenotype observed in *peo* mutants is not due to the absence of any of these proteins.

### Peo is a non-terminin protein that localizes to multiple chromosome sites

As pointed out in the introduction, terminin subunits are non-conserved fast-evolving proteins that localize and function only at telomeres. Peo is not a terminin-like protein because it does not share any of these properties. Peo is well conserved in *Drosophila* species (S3 Fig) and has homologues in mouse and humans (Ft1 and AKTIP, respectively). In addition, a three-dimensional model of *Drosophila* Peo is rather similar to an AKTIP model [43].

**Fig 8:**
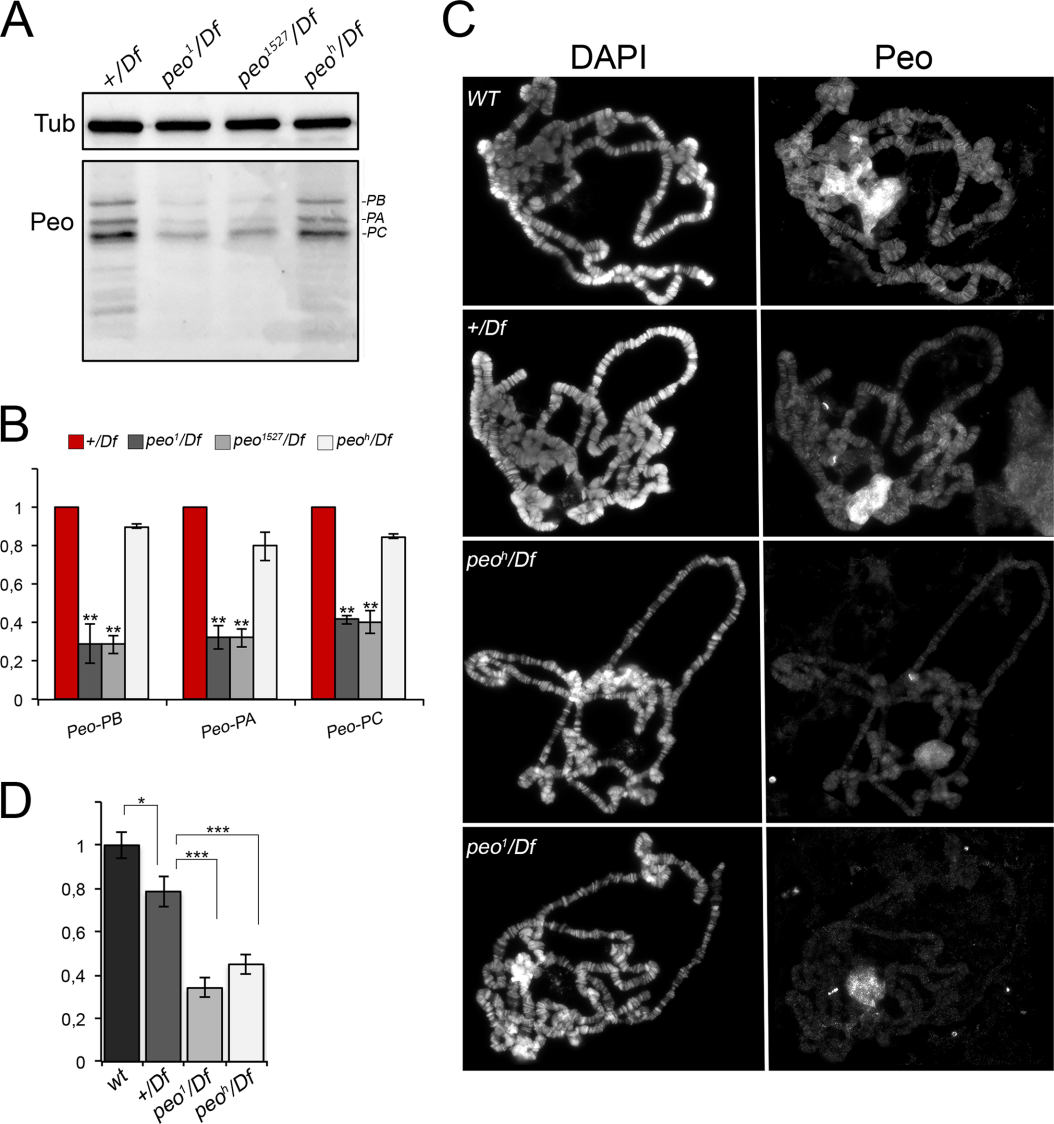
Peo expression and localization in polytene chromosomes. (A) Western blotting showing the levels Peo expression in larval brains. The affinity purified anti-Peo antibody reacts with 3 bands of the apparent molecular weights of 32, 28 and 25 kDa, which are likely to correspond to the 3 Peo isoforms (PA, PB and PC; see Fig 2 and FlyBase). (B) Quantification of the intensities of the 3 bands (see Materials and Methods) after normalization to the tubulin band (Tub) used as loading control; bars show the mean values of 3 experiments ± SEM. In *peo* ^*1*^ */Df* and *peo* ^*1527*^ */Df* brains the 3 bands are reduced by 60–70% compared to *+/Df* brains used as control; ** significantly different from *+/Df* with p < 0.01 in the Student's t test. In *peo* ^*h*^ */Df* brains the band intensity reduction is modest, ranging from 10 to 20%. (C) Immunostaining of wild type (WT), *+/Df*, *peo* ^*h*^ */Df* and *peo* ^*1*^ */Df* polytene chromosomes showing that Peo accumulates in the nucleolus and decorates many chromosome bands. (D) Fluorescence quantification (± SEM) of chromosome arms (but not of the nucleolus; see Materials and Methods) showed that the fluorescence intensities of *peo* ^*h*^ */Df* and *peo* ^*1*^ */Df* chromosomes are both significantly lower than those of either *+/Df* or wild type chromosomes. * and *** significant with p < 0.05 and p < 0.001 in the Student's t test, respectively.

To determine the subcellular localization of Peo we raised a rabbit polyclonal antibody against the entirety of Peo and affinity purified it against a GST-Peo fusion protein (see Material and Methods). Western blotting analysis showed that this antibody recognizes 3 bands of ~ 32, ~28 and ~ 25 kDa. In extracts from *peo* ^*1*^ */Df*, and *peo* ^*1527*^ */Df* larval brains, these 3 bands were reduced by approximately 70% compared to *+/Df* controls (Fig 8A and 8B). Thus, the band with the highest molecular weight is likely to correspond to the longest Peo isoform, while the other two bands might correspond to the shorter isoforms (see Fig 2). In *peo* ^*h*^ */Df* mutant brains, the Peo bands were reduced by approximately 20% with respect to *+/Df* brains, consistent with the relatively low TF frequency observed in *peo* ^*h*^ mutants (Fig 1). These results indicate that *peo* ^*1*^ and *peo* ^*1527*^ are strong hypomorphs compared to the weaker *peo* ^*h*^ allele.

Indirect immunofluorescence experiments on wild type polytene nuclei showed that the anti-Peo antibody stains the nucleolus and many bands along the polytene chromosomes (Fig 8C and 8D). In *peo* ^*h*^ */Df* and *peo* ^*1*^ */Df* nuclei, both the nucleolus and chromosome staining were significantly reduced compared to either *+/Df* or wild type nuclei, confirming the specificity of the antibody. In addition, the polytene chromosomes of *peo* ^*h*^ */Df* larvae were more intensely stained than those of *peo* ^*1*^ */Df* larvae (Fig 8C and 8D), confirming that the *peo* ^*1*^ allele is stronger than *peo* ^*h*^.

Although Peo is enriched at numerous polytene bands and interbands, we were not able to detect clear Peo accumulations at chromosome ends. Given that Peo interacts with terminin and it is required to prevent telomere fusion, the most likely explanation for this finding is that Peo is present at the telomere caps in amounts that are not detected by the antibody and the immunostaining technique used here.

### Peo is required for DNA replication

**Fig 9:**
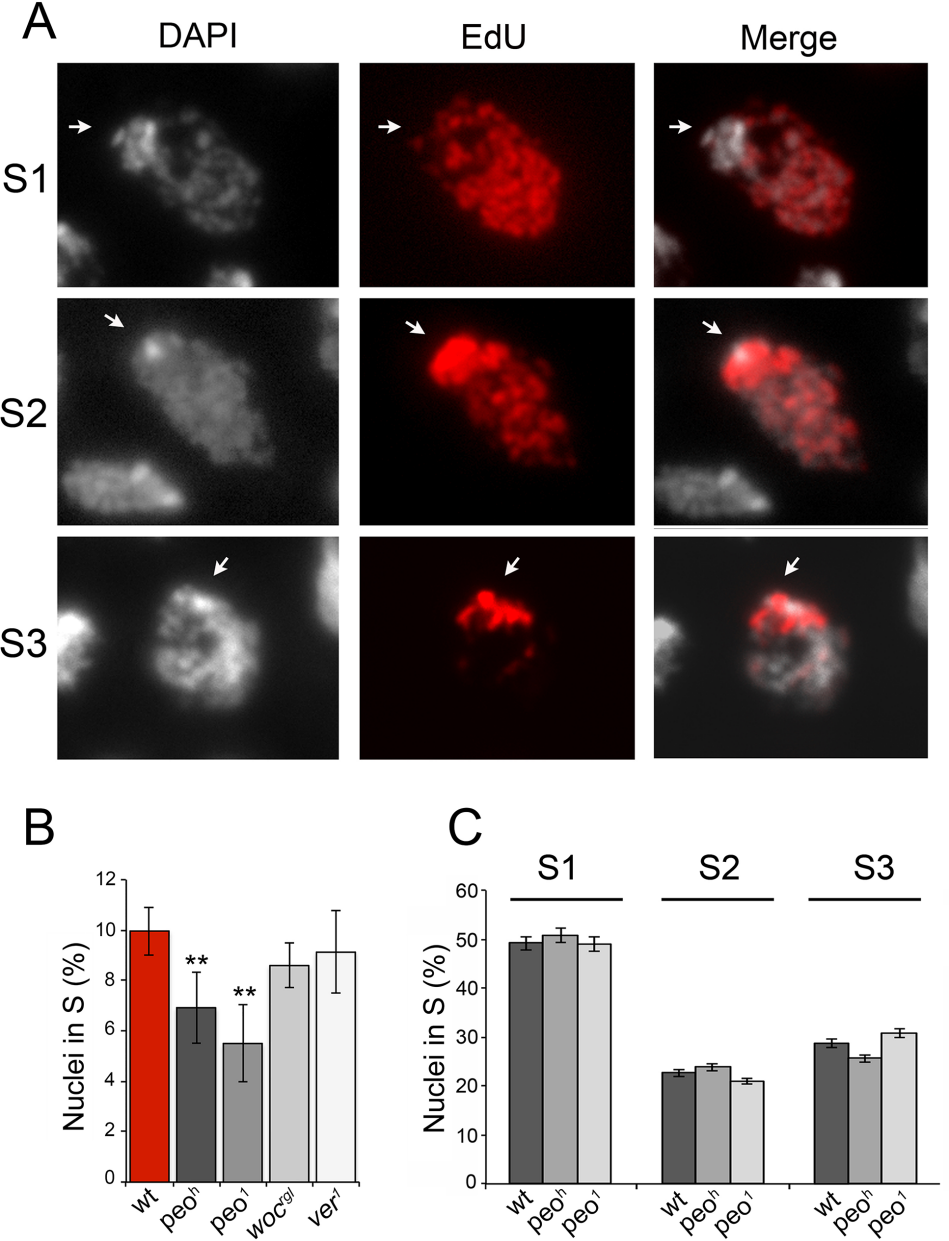
Mutations in *peo* affect DNA replication in brain cell nuclei. (A) Analysis of EdU-treated brain cell nuclei reveal 3 gross incorporation patterns: S1 nuclei incorporate EdU in most of the nucleus but not in the DAPI stained chromocenter and are presumably in early S; S2 nuclei incorporate EdU in both the chromocenter and the rest of the nucleus and are likely to be in mid-S; S3 nuclei incorporate EdU only in the chromocenter an are thus in late S. (B) *peo* mutant brains exhibit a significant reduction in the frequency of EdU positive nuclei compared to wild type (wt), *ver* or *woc* mutant brains (** significant in Student's t test with p <0.01). The frequencies of EdU positive nuclei in *ver* and *woc* brains are not significantly different from the wild type frequency (C) Mutations in *peo* do not alter the relative frequencies of S1, S2 and S3, nuclei suggesting that Peo is not required for a specific step of the S phase. The frequencies and types of EdU labeled nuclei in each sample (wild type, *peo* ^*h*^ */peo* ^*h*^ and *peo* ^*1*^ */peo* ^*1*^) were obtained by examining at least 5,000 nuclei from at least 6 brains.

We have recently found that AKTIP/Ft1 is required for proper telomeric DNA replication [43]. This finding suggested that *peo* mutations could also impair DNA replication and specifically affect heterochromatic telomeres, which are likely to replicate at the end of the S phase together with the bulk of heterochromatin (reviewed in [38]). To test this possibility we examined DNA replication in brain cells by analyzing the incorporation of the EdU (5-ethynyl-2’-deoxyuridine) analog of thymidine. Brains were incubated in saline containing 10 mM EdU for 1 h, immediately fixed and then stained with the Click-It Alexa Fluor method to detect EdU (see Methods). In wild type brains, 10% of the nuclei were actively replicating their DNA and incorporated EdU, whereas in *peo* ^*h*^ */peo* ^*h*^ and *peo* ^*1*^ */peo* ^*1*^ brains the frequency of EdU-labeled nuclei dropped to 7% and 5.5%, respectively (Fig 9A and 9B). In contrast, in *woc* and *ver* mutants, the frequency of EdU-positive nuclei was not significantly different from wild type controls, suggesting that the DNA replication defect observed in *peo* mutants is a specific outcome of the reduced *peo* activity and not a general consequence of impaired telomere protection.

EdU staining also allowed subdivision of the S phase according to the incorporation pattern. We distinguished nuclei in early/mid S (S1) in which the nucleus was partially or completely stained with the exception of the heterochromatic chromocenter, nuclei in mid/late S (S2) in which both the chromocenter and the less compact nuclear areas were stained, and nuclei in late S (S3) in which only the chromocenter displayed EdU incorporation (Fig 9). We found that wild type and *peo* mutant brains do not differ in the relative frequencies of nuclei showing S1, S2 and S3 EdU incorporation patterns. Thus, we conclude that the wild type function of *peo* is required for general DNA synthesis and not for completion of specific sub-phases of DNA replication.

**Fig 10:**
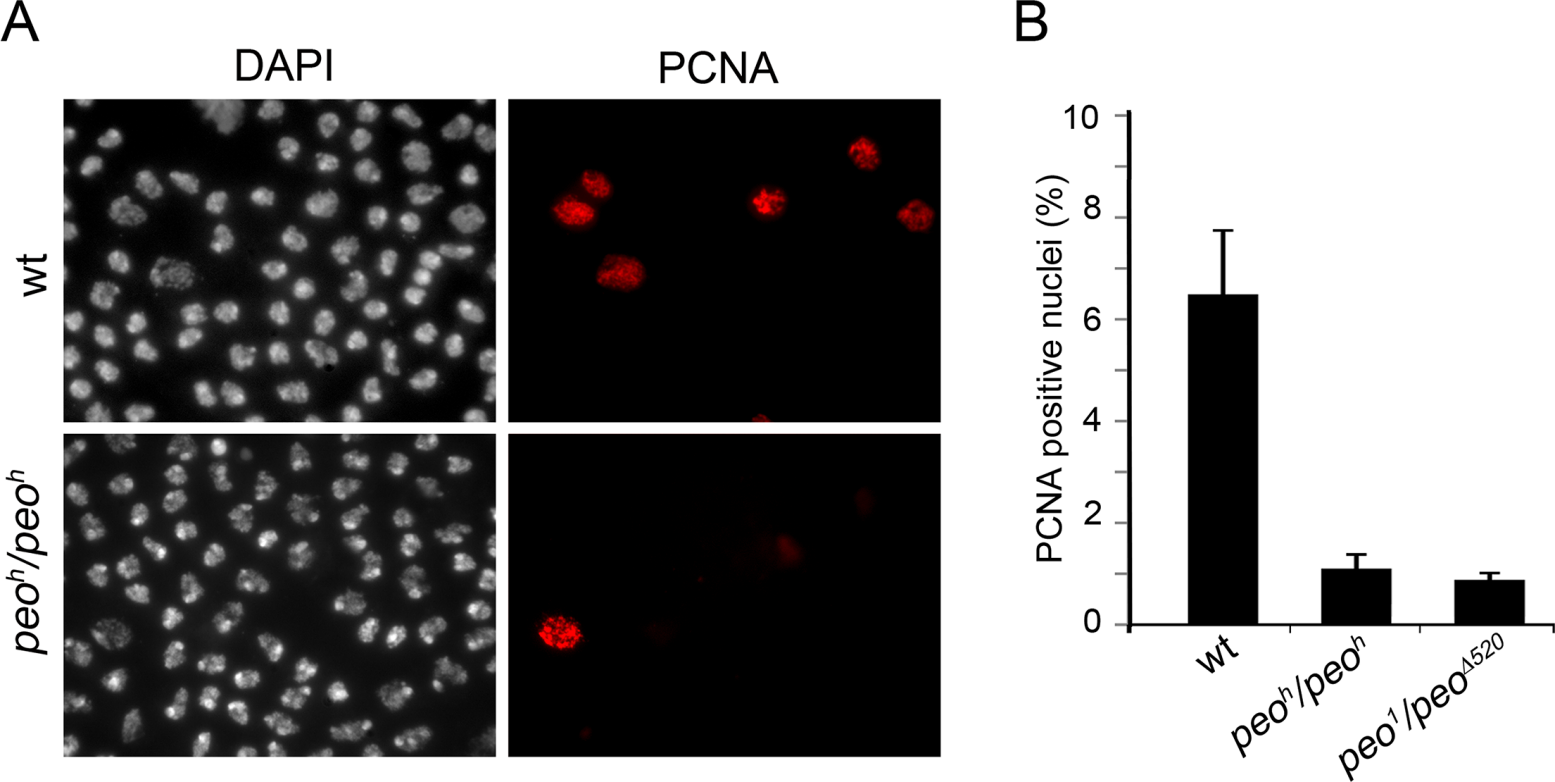
Peo is required for PCNA incorporation into brain cell nuclei. (A) Example of brain cell nuclei stained for PCNA after extraction with Triton X-100. (B) Frequencies of PCNA positive nuclei. The frequencies of PCNA positive nuclei in each sample (wild type, *peo* ^*h*^ */peo* ^*h*^ and *peo* ^*1*^ */peo* ^*∆520*^) were obtained by examining at least 4000 nuclei from at least 4 brains.

To gather additional information on the role of Peo in DNA replication we analyzed the distribution of PCNA (proliferating cell nuclear antigen) in *peo* ^*1*^ */peo* ^*1*^ and *peo* ^*h*^ */peo* ^*h*^ mutant nuclei. PCNA is a processivity factor for DNA polymerases; in nuclei PCNA is either present in a soluble form that can be extracted by detergent treatment or in a detergent-resistant form tightly associated with DNA replication forks (chromatin-bound PCNA) [44]. The analysis of Triton X-extracted brain preparations immunostained for PCNA showed that in wild type 7% of the nuclei were PCNA-positive, while in *peo* ^*1*^ */peo* ^*∆520*^ and *peo* ^*h*^ */peo* ^*h*^ brains the frequencies of PCNA-stained nuclei were 1.2% and 0.8%, respectively (Fig 10). These results are consistent with those on EdU incorporation and show that in *peo* mutants the frequency of nuclei with chromatin-bound PCNA is much lower than in control.

### A general impairment of DNA replication does not result in TFs

**Fig 11:**
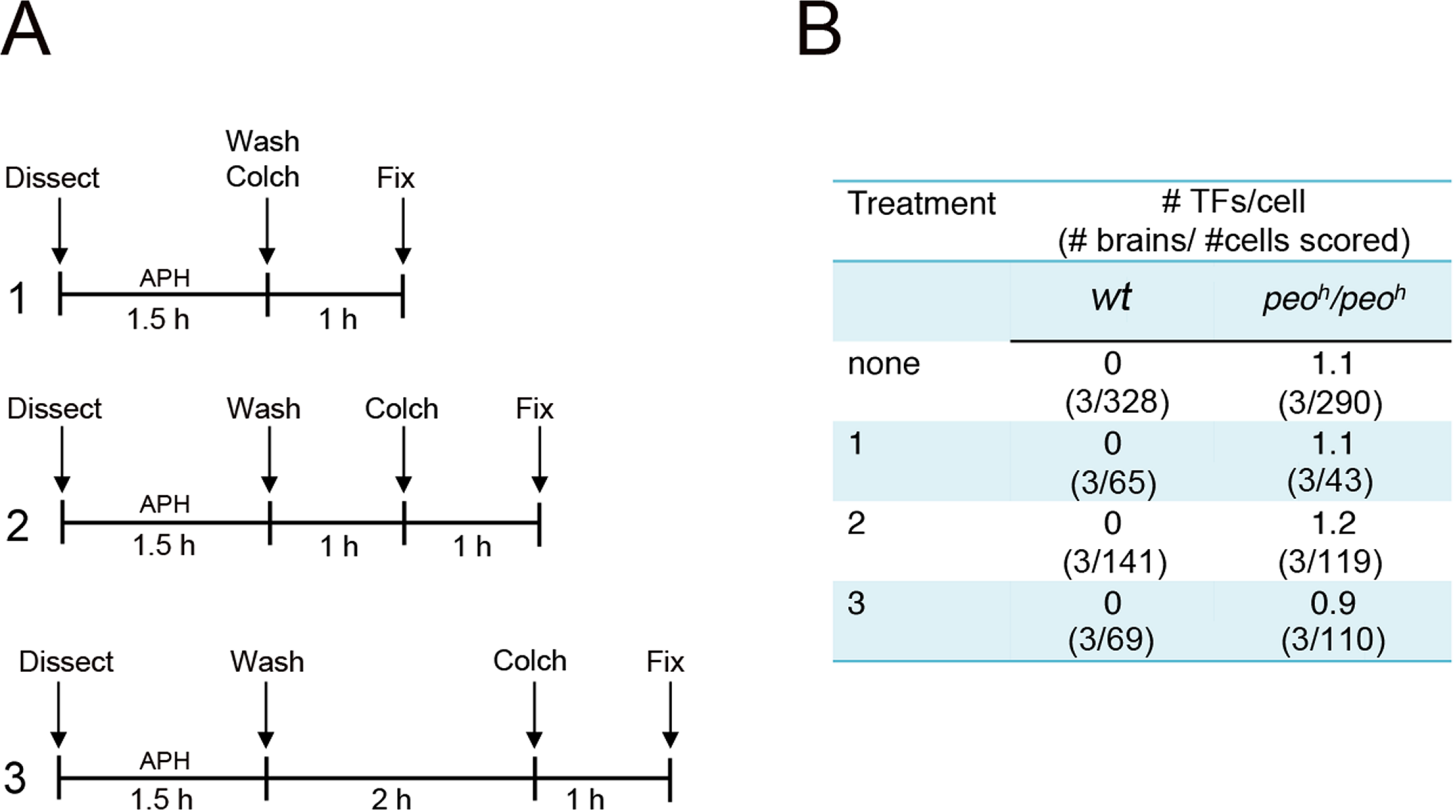
Aphidicolin (APH) does not induce TFs in *Drosophila* brain cells. (A) Experimental scheme used. Larval brains were dissected in saline (Dissect), incubated for 1.5 hours in saline plus APH, briefly rinsed in saline (Wash), and then transferred to 3 ml of APH-free Schneider’s medium supplemented with 10% FBS; Colch, addition of colchicine; Fix, fixation. Wild type and *peo* ^*h*^ */peo* ^*h*^ control brains were not treated with APH but processed like the APH-treated brains; they were fixed after 2 hours incubation in the medium following colchicine addition. (B) Results of the experiments outlined in A.

Collectively our results suggest three possibilities: (i) that any impairment of *Drosophila* telomere replication results in telomere fusion, (ii) that Peo is required for a specific step of telomere replication and that an incorrect execution of this steps results in fusigenic lesions, or (iii) that Peo plays a dual function being independently required for telomere replication and telomere capping. To discriminate between these possibilities we treated wild type and *peo* ^*h*^ */peo* ^*h*^ mutant brains with the DNA polymerase inhibitor aphidicolin (APH), which is known to impair human telomere replication [45–47]. As shown in Fig 11, control and mutant brains were treated in three different ways. Dissected brains were incubated for 1.5 hours in 110 mM APH in NaCl 0.7%. They were then washed, transferred into 3 ml of APH-free saline and fixed 1, 2 or 3 hours after the end of the APH treatment; in all cases, 1 hour before fixation, we added colchicine to the saline to collect metaphases. None of the APH treatments caused TFs in wild type brains or increased the TF frequency in *peo* ^*h*^ */peo* ^*h*^ mutants. We note that the APH treatment was highly effective as it caused a strong reduction in the frequency of mitoses in brains fixed 1 h after the end of the APH treatment (Fig 11B).

Our previous work has shown that mutations in Drosophila *timeless2* (*tim2*) result in chromosome breakage but not in TFs [48]. Tim2 is the *Drosophila* ortholog of mammalian TIM, a replisome component that facilitates DNA synthesis [49, 50] and is required for telomere replication [8]. These results prompted us to examine the cytological phenotype of brains from mutants in the *Blm* (formerly *mus-309*) gene [51], the *Drosophila* homolog of the Bloom syndrome gene, which encodes a DNA helicase required for mammalian telomere replication [46, 47]. We found that *Blm* ^*D2*^ */Blm* ^*D3*^ mutant brains exhibit chromosome breaks ranging from 4 to 7% but not TFs (500 metaphases scored from 7 brains). These results suggest that a general impairment of *Drosophila* telomere replication does not result in TFs.

## Discussion

### Mutations in *peo* preferentially affect specific *Drosophila* telomeres

We have shown that strong *peo* mutants exhibit an average of 5 TFs/cell; these TFs appear to involve all the telomeres of the *Drosophila* chromosome complement making difficult a reliable assessment of the relative involvement of individual telomeres in fusion events. However, in weak *peo* mutants or in strong mutants bearing a *peo* ^*+*^ rescue construct, which exhibit ~ 1 TF /cell, the majority of fusions involved the XR, the Y and the 4^th^ chromosome telomeres. These results suggest that these telomeres are preferentially affected by mutations in *peo* and that this effect is partially masked in strong mutants where most telomeres are fused.

In all the other *Drosophila* mutants we characterized (*eff*, *Su(var)205*, *cav*, *mre11*, *rad50*, *nbs*, *tefu*, *woc*, *moi* and *ver*) individual telomeres were engaged in TFs with frequencies that are different from those expected for a random involvement. The telomeres of the major autosomes were involved in TFs more frequently than expected, while participation of the XL telomere in fusions was either slightly lower or conformed to the expected frequency. In contrast, the Y, the XR, and the 4^th^ chromosome telomeres were engaged in TF less than expected (Fig 3 and S1 Table). *peo* mutants displayed an inverse TF pattern, with higher than expected frequencies of fusions involving the Y, the XR, and the 4^th^ chromosome telomeres (Fig 3 and S1 Table). To the best of our knowledge, this is the first case in which individual telomeres of an organism exhibit different fusigenic capabilities in response to the genetic background. We believe that this finding reflects the peculiar structural differences between the telomeric regions of the different *Drosophila* chromosomes.

**Fig 12:**
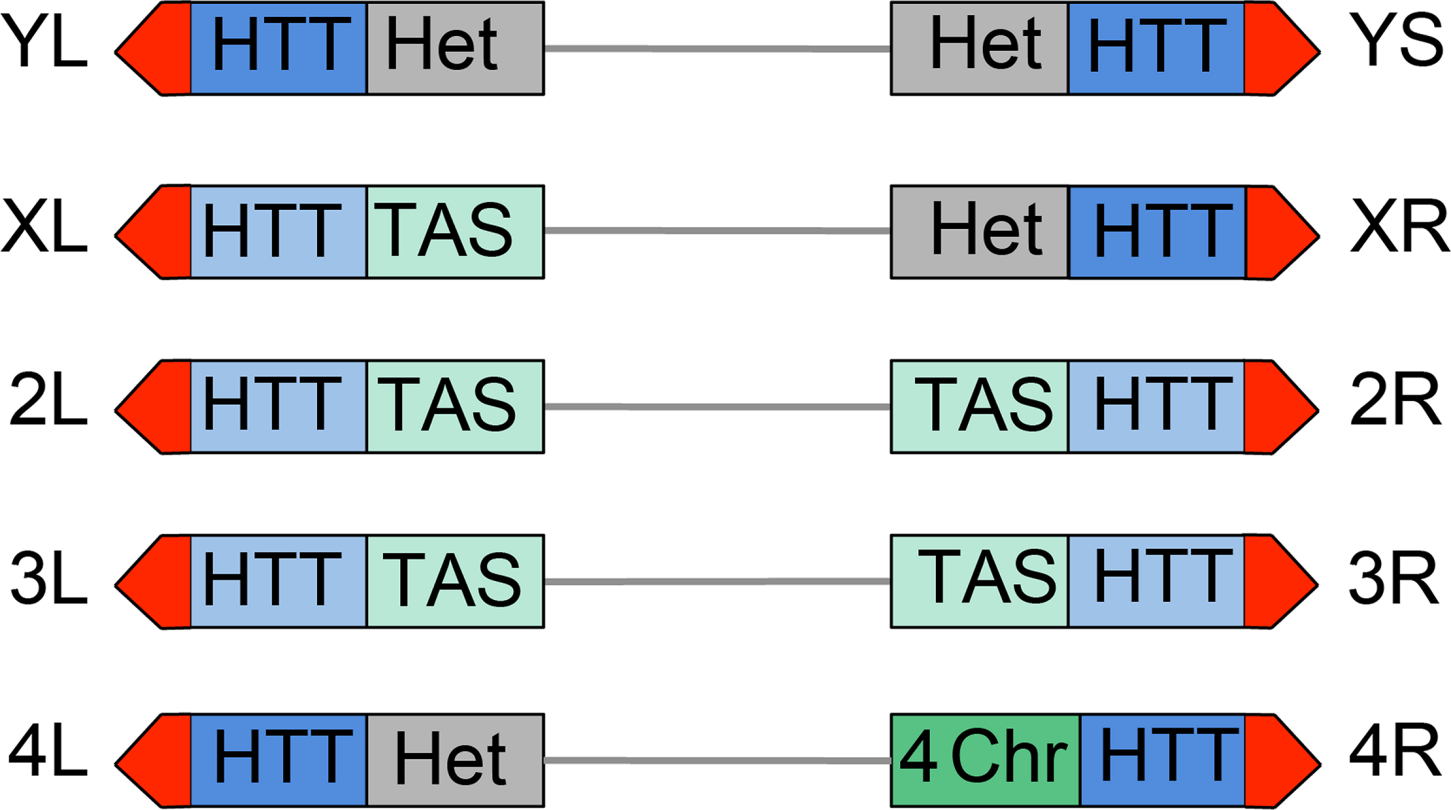
Structure of *Drosophila* telomeres and subtelomeres. Schematic representation of *Drosophila* chromosome ends. From a distal to a proximal direction chromosome ends contain the terminin-associated region (red) that may extend for approximately 10 Kb (22), the HTT array that vary in length from 26 to 147 Kb [81] and may either repress (dark blue) or not repress (light blue) the expression of transgenes; the TAS sequences (light green) that comprise about 20 Kb [55, 81], or different types of chromatin: constitutive heterochromatin (Het, grey), or the 4^th^ chromosome chromatin (4-ch, dark green) that has distinctive properties as well as properties shared by euchromatin and heterochromatin. The repressive properties of the YL, XR and 4L HTT have been inferred from those of the YS HTT; the properties of XL and 2L HTT are inferred from those 2L, 3L and 3R HTT. See text for detailed explanation.

All *Drosophila* chromosomes of wild type strains terminate with HTT arrays of variable length (made of complete and incomplete *HeT-A*, *TART* and *TAHRE* elements) (reviewed in [5, 52, 53]) and are capped by the multiprotein terminin complex (reviewed in [6]). However *Drosophila* telomeres differ from each other in both the type of subtelomeric chromatin and in the properties of their HTT arrays (Fig 12). One of the most straightforward features that contradistinguish some of the *Drosophila* telomeres is their association with constitutive hetrochromatin. Approximately one-third of the *Drosophila* genome is made of constitutive heterochromatin; the entire Y chromosome, the short arm (XR) and the proximal 40% of the long arm (XL) of the X chromosome, the short arm (4L) and the proximal 70% of the long arm (4R) of the 4^th^ chromosome, and the centric 25% of chromosomes 2 and 3 are heterochromatic [38, 54]. Thus the HTT arrays of YL, YS, XR and 4L are linked to constitutive heterochromatin (Fig 12). The HHT blocks of XL and those of the major autosomes are not directly associated with euchromatin but are instead juxtaposed to divergent clusters of subtelomeric repeats, known as telomere associated sequences (TAS) (reviewed in [55]). The TAS are not only different in sequence but are also occasionally absent from the subtelomeric regions, suggesting that their presence is not essential for proper telomere function [55]. Finally, the 4R telomere is joined to a special type of chromatin that has peculiar features, as well as features shared with both euchromatin and heterochromatin; for example the 4R distal chromatin is enriched in the 4th chromosome-specific Painting of four (Pof) protein and the heterochromatic markers HP1a and histone 3 methylated at lysine 9 (H3K9) (reviewed in [56]) (Fig 12).

Additional features that differentiate *Drosophila* telomeres are the silencing properties of their HTT arrays (Fig 12). Several studies have shown that *white* ^*+*^ transgenes inserted within or next to the TAS are partially silenced leading to a variegated eye phenotype (a phenomenon known as telomere position effect or TPE) (reviewed in [5]). However, *white* ^*+*^ transgenes inserted into the HTT arrays behave differently depending on the insertion site. Insertions into the HTTs of telomeres not directly joined to heterochromatin such as those of the 2L, 3L and 3R arms do not appear to be subject to TPE [57]. In contrast *white* ^*+*^ transgenes inserted within the 4R (only one tested) and YS (6 tested) HTTs lead to a variegated eye phenotype [57, 58]. Furthermore, the *white* ^*+*^ transgenes inserted into the HTT array of YS and those embedded into or near the TAS respond differently to genetic modifiers. For example, mutations in *Su(var)205* (HP1a), which suppress variegation of genes relocated next to constitutive heterochromatin (position effect variegation or PEV; reviewed in [59, 60]) do not affect the TAS-associated TPE [5, 55] but strongly suppress the TPE of transgenes inserted into the HTT of YS (58). These results indicate that the chromatin of the YS HTT shares some features with constitutive heterochromatin and has different properties from the chromatin that includes the autosomal HTT arrays and the TAS.

These results suggest that the HTT arrays of XR, YL, and 4^th^ chromosome telomeres have properties similar to those of YS, namely they are subject to a “heterochromatinization” process related to their particular location. Numerous studies on PEV have shown that proteins that are typically enriched in the heterochromatin can spread into neighboring euchromatic genes changing their chromatin composition and packaging and downregulating their expression [59, 60]. Thus, although the properties of the HTT arrays of YL, XR and 4^th^ chromosome have not been tested, it is quite likely that they are similar to those of YS. This would suggest that in a *peo* mutant background the preferential involvement of these telomeres in TF is a consequence of their “heterochromatinization”. This in turn implies that the heterochromatic markers of the HTT regions of the YL, XR and 4h chromosome telomeres extend to the terminin-coated chromosome ends. However, “heterochromatinization” does not extend to the HTT arrays at the end of the left arm end of the *B* ^*S*^ *w* ^*+*^ *y* ^*+*^ *Y* chromosome, because in *peo* mutants this marked Y forms much fewer Y rings than a normal Y. Our findings on ring Y formation in *peo* mutants also suggest that the different “heterochromatic” telomeres respond differently to Peo depletion. The higher than expected frequency of ring Ys observed in *peo* mutants could be a consequence of the particularly high fusigenic properties of the Y telomeres.

We would like to note that the “heterochromatinization” of the YL, YS, XR and 4^th^ chromosome termini is the natural condition of these telomeres and that we only know that this condition makes them more fusigenic than the other *Drosophila* telomeres. However, we have no information on the actual properties of these telomeres. Namely, we do not know the properties of their terminin-associated regions. For example, we do not know whether the terminin-coated chromosome ends of YL, YS, XR and 4L have different silencing properties and different responses to PEV and TPE modifiers compared to the terminin-bound regions of the other chromosome ends.

### The mechanism underlying TF formation in *peo* mutants

We have shown that mutations in *peo* genetically interact with mutation in genes that encode the terminin subunits. Consistent with these results, we have also shown that Peo directly binds terminin and mapped the Peo-HOAP interacting domains. Thus, although Peo does not share the properties of the terminin proteins, it is clearly a component of the *Drosophila* telomere capping machinery. What is then the role of *peo* in telomere protection? The finding that mutations in *peo* do not affect HOAP, Moi and Ver localization at telomeres strongly suggest that loss of *peo* function does not cause TFs by affecting terminin recruitment at telomeres. Similarly, the normal accumulation of HP1a at the telomeres of *peo* mutants suggests a telomere fusion mechanism independent of HP1a. More in general, the fact that *peo*, but not the other TF genes (*eff*, *Su(var)205*, *cav*, *mre11*, *rad50*, *nbs*, *woc*, *moi* and *ver*), preferentially affect “heterochromatic” telomeres suggests that *peo* might either act upstream to these genes or function in a telomere protection pathway that does not involve them.

The findings that mutations in *peo* impair DNA replication and preferentially affect late replicating “heterochromatic” telomeres raise the possibility that defective telomere duplication might be fusigenic in *Drosophila*. However, there are several reasons that lead us to exclude this possibility. Should it be correct, one would expect that impairment of some of the many factors that mediate DNA replication would results in TFs. However, in addition to the aphidicolin treatment and *Blm* mutations described here, several additional DNA replication factors have been described whose loss fails to induce TFs. Hydroxyurea (HU), which blocks DNA replication by inducing a deoxyribonucleotide triphosphate (dNTP) pool depletion, does not induce TFs in brain cells [48]. In addition, a number of mutations (or RNAi treatments) disrupting different aspect of DNA replication do not results in TFs in *Drosophila* brain cells or S2 tissue culture cells, although they cause more or less extensive chromosome breakage. These include lesions in the genes/RNAs encoding the origin recognition (ORC) and the minichromosome maintenance (MCM) prereplication complexes, DNA primase, the Cul4 replication licensing factor, the replisome components DNA polymerase alpha, Rpa70, Tim2 and PCNA, as well as the chromatin assembly factor Caf1 that assists in loading the histone tetramer after DNA replication [21, 48, 61–64]. We thus hypothesize that the *peo*-dependent TFs are generated by a peculiar defect in telomeric DNA replication that creates specific fusigenic lesions. Heterochromatin replication is likely to be different from that of euchromatin, as it requires not only DNA duplication but also reinstallment of specific epigenetic markers such as heterochromatin-associated proteins and histone modifications (reviewed in [65]). It is thus possible that in the presence of weak *peo* mutations replication of “heterochromatic” telomeres is preferentially affected leading to specific Peo-dependent fusigenic lesions concentrated in the XR, the Y and the 4^th^ chromosome ends. In strong *peo* mutants, these fusigenic lesions would be generated also in “euchromatic” telomeres resulting into a more general involvement of telomeres in fusion events. However, we cannot exclude the possibility that *peo* plays a dual function being independently required for DNA replication and telomere capping.

In conclusion, we propose that Peo is a *Drosophila* telomere-capping protein that preferentially protects chromosome ends associated with heterochromatic markers. Our results also indicate that Peo is required for general DNA replication and most likely also for telomere replication. However, while it is possible that loss of Peo generates specific fusigenic lesions during telomere replication, it is unlikely that a general impairment of *Drosophila* telomere duplication leads to telomere fusion. In this respect *Drosophila* telomeres are similar to human telomeres, which fail to fuse following defective replication [8, 46, 47, 66].

## Materials and Methods

### *Drosophila* strains

The *pendolino* ^*1*^ *(peo* ^*1*^ *)* mutation was isolated by a cytological screen of 120 late lethal mutants mapping to the second chromosome, recovered after I element mobilization by I-R dysgenic crosses [67]. The late lethal mutations *l(2)1527*, *l(2)2723*, the insertion lines *p112*, *p221* and *p520* were described previously [30] and were kindly provided by P. Taghert (Washington University, MO). The *peo* ^*h*^ allele was isolated from a cytological screen of a collection of 193 late lethal mutants that arose in the Zucker’s collection of heavily mutagenized viable lines [68]. The *Df(2R)X3*, *Df(2R)B5*, *Df(2R)X1* and *Df(2R)BSC298* deletions, the insertion line *cbx* ^*05704*^, were obtained from the Bloomington Stock Center and are described in FlyBase. All *peo* alleles and the deficiencies of 2R were balanced over *CyO Tb* balancer [69]. The *eff*, *Su(var)205*, *cav*, *mre11*, *rad50*, *nbs*, *woc*, *moi* and *ver* mutations were previously described [10, 12, 15, 16, 21, 23, 24]. The DNA sequences flanking the *peo* ^*p112*^ insertion were obtained by inverse PCR using standard procedures.

To obtain the rescue constructs, the LD08052 full lenght *peo* cDNA was cloned into *pCASPER4* transformation vectors. Germline transformation was carried out by the BestGene Company. A *pCasper4 [w* ^*+*^, *peo* ^*+*^ *]* insertion (with a *Hsp70* promoter) on the X chromosome was used to establish the *[w* ^*+*^, *peo* ^*+*^ *]; peo* ^*1*^ */CyO Tb* stock. Animals from this stock were heat-shocked for 1h at 37°C every day starting from the embryonic stages; we then examined the brains of non-*Tb* third instar larvae for the presence and frequency of TFs and the adult flies for the presence of *w* ^*+*^, *peo* ^*+*^ *; peo* ^*1*^ */peo* ^*1*^ individuals. For the rescue experiments we also employed a *pCASPER4 [w* ^*+*^, *peo* ^*+*^ *-HA]* insertion (with a *Tubulin* promoter) on the third chromosomes. We established *[w* ^*+*^, *peo* ^*+*^ *-HA]*/*TM6C; peo* ^*1*^ */CyO Tb* and *[w* ^*+*^, *peo* ^*+*^ *-HA]*/*TM6C; peo* ^*h*^ */CyO Tb* stocks and examined them for TFs in non-*Tb* larvae. The *[w* ^*+*^, *peo* ^*+*^ *-HA]*/*TM6C; peo* ^*1*^ */CyO Tb* stock was also examined for the presence of *peo* ^*1*^ */peo* ^*1*^ non *Cy* flies. Information on the genetic markers and balancers used in this study is available at FlyBase (http://flybase.bio.indiana.edu/). Stocks were maintained and crosses were made on standard *Drosophila* medium at 25°C.

### Anti-Peo antibody production and purification

To generate anti-Peo polyclonal antibodies, rabbits were immunized with bacterially expressed 6His-Peo. Immunization and production of anti-Peo antibodies were carried out by the Agro-Bio Company (France).

To purify the anti-Peo antibody we used a bacterially expressed GST-Peo protein. About 1 mg of this tagged protein was run on a polyacrylamide gel and then blotted onto a nitrocellulose membrane. The membrane strip containing GST-Peo was cut out, washed in 100 mM glycine/HCl pH 2.5 for 5 min, washed for 5 min in TBS, blocked by incubation with 3% BSA for 1 hour, and washed again for 4 min in TBS. The membrane was then cut into small pieces and incubated overnight with rotation at 4°C in 2 ml of serum diluted 1:5 in TBS. After centrifugation and removal of supernatant the membrane pieces were washed for 15 min in 50 mM Tris/HCl pH 7.5, 500 mM NaCl, for 5 min in 50 mM Tris/HCl pH 7.5, 100 mM NaCl, and for 10 min in TBS. The membrane was then incubated for 30 min in 1ml of 100 mM glycine/HCl pH 2.5 to elute the antibody. The eluate was then mixed to a proper volume 1M Tris pH 8.8 to bring the final pH to 8.0, and then kept at 4°C before use.

### Chromosome cytology and immunostaining

DAPI-stained colchicine-treated larval brain chromosomes were prepared according to [10]. Preparation and immunostaining of mitotic and polytene chromosomes were carried out as described previously [10, 20], with minor modifications.

For anti-PCNA immunostaining, dissected larval brains were incubated in PBS with 0.5% Triton X-100 for 3 min, and then fixed in 3.7% formaldehyde according to [70]. Before immunostaining, brain squash preparations were Triton-X extracted by incubating the slides in 0.1% Triton X-100 containing PBS (PBT), 2 times for 10 min.

The primary antibodies used for immunostaining were: the rabbit anti-Peo described above diluted 1:10; rabbit anti-HOAP (1:100), mouse anti-HP1a (C1A9; 1:10), and mouse anti-PCNA (1:20; Abcam ab29). The anti-HP1a antibody C1A9 was obtained from the Developmental Studies Hybridoma Bank, created by the NICHD of the NIH and maintained at The University of Iowa, Department of Biology, Iowa City, IA 52242. After an overnight incubation at 4°C with the primary antibody, slides were washed twice in TBS-Tween 0.1% for 15 min and then incubated for 2 h at room temperature with FITC-conjugated goat anti-mouse (1:20; Jackson Laboratories), or AlexaFluor 555-conjugated donkey anti-rabbit (1:200; Invitrogen) antibodies. All slides were then mounted in Vectashield medium H-1200 with DAPI to stain DNA. *in vivo* detection and immunostaining of GFP-tagged proteins on polytene chromosomes were carried out as previously described [24].Chromosome preparations were analyzed using a Zeiss Axioplan epifluorescence microscope (CarlZeiss, Obezkochen, Germany), equipped with a cooled CCD camera (CoolSnap HQ, Photometrics, Woburn, MA). Gray-scale digital images were collected separately, converted to Photoshop format, pseudocolored, and merged.

To quantify the polytene chromosome fluorescence intensity after Peo immunostaining, we used the ImageJ software (National Institute of Mental Health, Bethesda, Maryland, USA). Given that the distribution of fluorescent bands along the chromosomes was rather uniform, for each polytene nucleus we selected 3–4 different chromosome regions of similar length, and measured both their fluorescence and the fluorescence of a close chromosome-free region to correct for background fluorescence. For each genotype (wild type, *+/Df*, *peo* ^*h*^ */Df* and *peo* ^*1*^ */Df*) shown in Fig 8 we measured at least 30 polytene regions from at least 10 nuclei.

### Yeast two-hybrid assay

The coding sequences of Peo, Hp1a and HOAP were PCR-amplified and cloned into pGBKT7 or pGAD-T7 vectors (Clontech). The *S*. *cerevisiae* AH109 strain was transformed with the indicated combinations of plasmids and assayed for growth on SD/–His/–Trp/–Leu selection plates supplemented with 20 mM 3-amino-1,2,4-triazole (3-AT), according to the manufacturer’s instructions.

### Purification of recombinant proteins

To obtain the GST-Moi, GST-Ver, GST-HOAP and GST-Peo fusion proteins, the corresponding full length cDNAs were cloned in either pGEX-6P1 or pGEX-3X, expressed in bacteria and purified as described previously [24]. The GST-Peo1, GST-Peo2, GST-Peo3, GST-HOAP ∆3, GST-HOAP ∆2, 3, GST-HOAP ∆1–3 and GST-HOAP ∆N-TERM truncated proteins were obtained by cloning the corresponding PCR-generated sequences in pGEX-6P1; bacterially expressed GST fusion proteins were then purified by incubating crude lysates with glutathione sepharose beads (QIAGEN) as recommended by the manufacturer. To generate 6His-Peo, the LD08052 *peo* full-length cDNA was cloned into the pQE32 expression vector (QIAGEN), and expressed in bacteria; 6His-Peo was affinity purified with a Ni-NTA resin using standard procedures.

### Protein extract preparation, GST pulldown and western blot

To obtain extracts for Western Blot analysis, 50 dissected third instar larval brains were lysed in an ice-cold buffer containing 20 mM Hepes KOH pH 7.9, 1.5 mM MgCl_2,_ 10 mM KCl, 420 mM NaCl, 30 mM NaF, 0.2 mM Na_3_VO_4,_ 25 mM BGP, 0.5 M PMSF, 0.1% NP40, and 1X protease inhibitor cocktail (Roche). To obtain HOAP-FLAG, Ver-FLAG and Peo-FLAG expressing S2 cells, *cav*, *ver* o*r peo* cDNAs were cloned in the *pAWF* vector (DGRC) in frame with the FLAG-coding sequence. For the expression of Moi-HA, the *moi* full lenght cDNA was fused in frame with the HA-coding sequence and then cloned into a *pCASPER4* vector. All constructs were transfected in S2 tissue culture cells using Cellfectin (Invitrogen), and cells were harvested 72 h after transfection. Extracts were lysed in 20 mM Tris pH 8.0, 420 mM NaCl, 1 mM MgCl_2_, 1 mM DTT, 0.1% NP40, and 1X protease inhibitor cocktail (Roche). For preparation of human cell extracts, HeLa cells expressing Peo-FLAG cloned in the pCDNA vector were harvested after 72hr transfection and lysated in 20 mM Tris pH 8.0, 420 mM NaCl, 1 mM MgCl_2_, 1 mM DTT, 0.1% NP40 and 1X protease inhibitor cocktail (Roche).

For GST-pulldown assays, protein extracts were incubated with 2 μg of each GST fusion protein bound to sepharose beads in a buffer containing 20 mM Hepes KOH, 20 mM NaF and 0.8% NP40 for 1h at 4°C. Sepharose-bound GST proteins were collected by centrifugation, washed several times with 20 mM Hepes KOH, 20 mM NaF and 1.8% NP40, and resuspended in Laemli buffer in a 30μl final volume for Western Blot analysis. For immunoblotting, protein samples were run into SDS polyacrilammide gels and electro-blotted on a nitrocellulose membrane (Bio-Rad) in a phosphate buffer (390 mM NaH_2_PO_4_ /610 mM Na_2_HPO4). For the detection of HOAP-FLAG, Ver- FLAG, Peo- FLAG, and Moi-HA, membranes were probed with anti-FLAG HRP-conjugated (1:1000; Roche), and anti-HA HRP-conjugated (1:500; Roche) antibodies; Peo and His-Peo were detected with our rabbit anti-Peo (1:100), and Giotto with our anti-Giotto (1:5000; [71]). Secondary antibodies were sheep anti-mouse IgG HRP-conjugated (1:5000), or donkey anti-rabbit IgG HRP-conjugated (1:5000) (both from Amersham Biosciences). The blots were developed using the ECL or ECL Plus method (Amersham Biosciences) and signals were detected with the ChemiDoc scanning system (BioRad). Band intensities were quantified using the image acquisition and analysis Image lab 4.0.1 software (Biorad).

### EdU incorporation and staining

EdU labeling was performed as per the manufacturer's instructions (Invitrogen, ClickiT Alexa Fluor 488 Imaging kit). Larval brains were cultured with 10μM EdU in 1 X PBS for 60 min prior to fixation and detection.

### Aphidicolin treatment

Brains from third instar larvae were dissected in saline (0.7% NaCl), incubated in saline with 110 μM Aphidicolin (APH) for 1.5 hours, rinsed in saline, and then transferred into a 33 mm Petri dish containing 3 ml of Schneider’s medium (SIGMA) supplemented with 10% fetal bovine serum (FBS, Gibco BRL) for 1, 2 or 3h. Colchicine at a final concentration of 10^-5^M was added to the medium 1 hour before fixation according to [10]. Control wild type and *peo* ^*h*^ */peo* ^*h*^ brains were incubated in saline without APH for 1.5, and processed like the APH-treated brains; they were fixed after 2 hours incubation n the medium.

### Bioinformatic analysis of the Peo structure

As a first step towards the construction of a three-dimensional model of Peo, we used its full-length sequence (Accession code: Q7K4V4) as a query to search the UniProtKB database (http://www.uniprot.org/) using CSI-BLAST [72], with an Expectation (E) value threshold of 10^–5^. Iterative searches of the database yielded 59 unique sequences. The retrieved sequences were aligned with the CLUSTALW software [73] with default parameters. The multiple sequence alignment (MSA) was next used as a seed to construct a Hidden Markov Model (HMM) of the family.

The HMM was employed to search the Pfam database (http://pfam.sanger.ac.uk/) via the HHpred server [74]. The highest scoring hit was the ubiquitin-conjugating enzyme (UBC) family also known as the E2 enzyme family [34] (probability to be a true positive more than to 99%, E-value equal to 1.2 x10^-42^). The third scoring hit was the ubiquitin E2 variant (UEV) family (Pfam ID: PF05743) that includes UBC homologs such as Tsg101, Mms2 and UEV1 (probability to be a true positive equal to 96.44%, E-value equal to 1.3 x10^-4^). Peo belongs to the UEV family because it contains an aspartic acid residue (at position 106, according to SwissProt numbering) in place of the E2 active site cysteine, and it is unable to catalyze ubiquitin transfer as it lacks the cysteine that forms a transient thioester bond with the C-terminus of ubiquitin (Ub).

Prediction of potentially disordered regions using the GeneSilico MetaDisorder server (http://iimcb.genesilico.pl/metadisorder/) revealed that at the C-terminus of Peo there is a stretch of ~ 70 aa (from residue 177 to 244, according SwissProt numbering) that has the tendency to be intrinsically disordered (i.e. lack a unique three dimensional structure at least in the absence of a binding partner), while the region including residues 16–176 shows propensity to form a folded globular domain with a well-defined pattern of secondary structures as revealed by the Quick2d web server analysis [75].

Because no homologous structure with sufficiently high sequence identity with Peo is available, we performed the Peo modeling using the composite approach implemented in I-TASSER server (Iterative Threading ASSEmbly Refinement) [76].The Peo sequence from residue 16 to 176 (predicted to fold in a globular domain) was submitted to the server and the model with the best confidence score (C-score = 0.5) returned by I-TASSER was selected. We added hydrogen atoms in this model using HAAD software [77] and refined it close to the native structure using FG-MD molecular dynamics based algorithm [78].

Our final refined model of Peo was evaluated as a potentially extremely good model (with a predicted LGscore of 2.50) by the PRO-Q model quality assessment program [79]. The QMEAN score [80] was 0.6 (the variability range is 0–1, with 1 being a perfect model). Collectively these parameters indicate that the Peo three-dimensional model is sufficiently accurate for making functional inferences.

## Supporting Information

S1 Table Patterns of telomere fusions (TFs) in different mutants defective in telomere protection. FTs, fused telomeres (each TF involves 2 pairs FTs); A, major autosomes, namely 2L, 2R, 3L and 3R arms; XL and XR, left and right arm of the X chromosome, respectively; 4th, fourth chromosomes, 4L and 4R arms; Y chromosome, YL and YS arms; Eu FTs, fused "euchromatic telomeres" (A and XL); Het FTs, fused "heterochromatic telomeres" (XR, 4th, and Y). The expected numbers of FTs have been calculated on the basis of the expected frequencies from a random involvement of telomeres in fusions events (Males, A, 50%; XL, 6.25%; XR, 6.25%; 4th, 25%; Y, 12.5%. Females, A, 50%; XL, 12.5%; XR, 12.5%; 4th, 25%). With the exception of those highlighted in blue, all the differences between the observed and expected numbers of FTs are statistically significant in the Chi-square test. The differences between the global numbers of Eu FTs and Het FTs are all significant with p< 0.001. In *peo* mutants, the numbers of all types of heterochromatic telomeres involved in fusion events are significantly higher than expected, while those of euchromatic telomeres are significantly lower. In *ver*, *cav*, *moi*, *Su(var)205*, *tefu*, *woc*, *nbs*, *mre11*, and *eff* mutants there is an opposite pattern of telomere fusions, with a few exceptions. The numbers highlighted in blue are not significantly different; those highlighted in yellow are significantly different, but the observed differences are at odds with the general pattern of telomere fusions observed in these mutants.
(DOCX)

S1 Fig *peo* interacts with HOAP in the yeast two-hybrid assay. Protein interactions between Peo and HOAP or HP1a were tested by the yeast two-hybrid assay. The GAL4 DNA-binding domain (BD) and GAL4 activation domain (AD) were fused to the indicated proteins. Cells expressing the indicated combinations of bait (BD) and prey (AD) fusion proteins were plated on medium lacking Leucine and Tryptophan (-LT). The presence of physical interactions is revealed by growth on plates lacking Histidine and supplemented with 3-AT (-LTH +20 mM 3-AT). The interaction between HP1a and HOAP was used as a positive control.
(TIF)

S2 Fig The predicted Peo structure and its comparison with those hUBC13, hUEV2, hUEV1 and AKTIP. (A) Alignment of the amino acid sequence of Peo, hUEV1, hUEV2, hUBC13 and AKTIP. Secondary structure elements predicted for Peo are shown above the alignment. Red and blue arrowheads indicate the sites of the catalytic Cys (Asp in Peo) and the HPN motif (HPH in Peo), respectively. The red broken line indicates the predicted intrinsically disordered portion of Peo. (B) A three-dimensional molecular model for Peo. The arrows point to the N terminus and to the starting site of the disordered C-terminal region (not depicted); the variant Asp residue and His-Pro-His motif are represented as sticks and indicated by red and purple arrows.
(TIF)

S3 Fig The *D*. *melanogaster* telomere-capping proteins exhibit different degrees of evolutionary conservation. The graph shows the percentages of identity between the *D*. *melanogaster* proteins and the homologous proteins form 11 *Drosophila* species (*D*. *mel*, *D*. *melanogaster; D*. *sim*, *D*. *simulans; D*. *sec*, *D*. *sechellia; D*. *yak*, *D*. *yakuba; D*. *ere*, *D*. *erecta; D*. *ana*, *D*. *ananassae*, *D*. *pse*, *D*. *pseudoobscura; D*. *per*, *D*. *persimilis; D*. *wil*, *D*. *willistoni; D*. *moj*, *D*. *mojavensis; D*. *vir*, *D*. *virilis; D*. *grim*, *D*. *grimshawi)*. The identity percentage is the percentage of matches between two amino acid sequences, calculated using the pairwise alignment EMBOSS Needle Software. Note that the terminin components HOAP, Moi, Ver and HipHop are poorly conserved, whereas Peo exhibits a high degree of conservation comparable to that of nonterminin proteins such as Woc or HP1a.
(TIF)
