# Supplementary material for: The Analysis of Pendolino (peo) Mutants Reveals Differences in the Fusigenic Potential among Drosophila Telomeres
Source: PLoS Genet. 2015 Jun 25;11(6):e1005260. doi: 10.1371/journal.pgen.1005260 (PMC4481407; doi:10.1371/journal.pgen.1005260)
Supplement: S1 Table — FTs, fused telomeres (each TF involves 2 pairs FTs); A, major autosomes, namely 2L, 2R, 3L and 3R arms; XL and XR, left and right arm of the X chromosome, respectively; 4th, fourth chromosomes, 4L and 4R arms; Y chromosome, YL and YS arms; Eu FTs, fused "euchromatic telomeres" (A and XL); Het FTs, fused "heterochromatic telomeres" (XR, 4th, and Y). The expected numbers of FTs have been calculated on the basis of the expected frequencies from a random involvement of telomeres in fusions events (Males, A, 50%; XL, 6.25%; XR, 6.25%; 4th, 25%; Y, 12.5%. Females, A, 50%; XL, 12.5%; XR, 12.5%; 4th, 25%). With the exception of those highlighted in blue, all the differences between the observed and expected numbers of FTs are statistically significant in the Chi-square test. The differences between the global numbers of Eu FTs and Het FTs are all significant with p< 0.001. In peo mutants, the numbers of all types of heterochromatic telomeres involved in fusion events are significantly higher than expected, while those of euchromatic telomeres are significantly lower. In ver, cav, moi, Su(var)205, tefu, woc, nbs, mre11, and eff mutants there is an opposite pattern of telomere fusions, with a few exceptions. The numbers highlighted in blue are not significantly different; those highlighted in yellow are significantly different, but the observed differences are at odds with the general pattern of telomere fusions observed in these mutants. (DOCX) [file pgen.1005260.s001.docx]

**S1 Table. Patterns of telomere fusions (TFs) in mutants defective in telomere protection**

| **Genotype**  **(TF frequencies)** | **Sex** |  | **# Fused Telomeres (FTs)** | | | | | | | |
| --- | --- | --- | --- | --- | --- | --- | --- | --- | --- | --- |
|  |  | **Total** |  | **A** | **XL** | **XR** | **4th** | **Y** | **# Eu** | **# Het** |
| ***ver^1^/ver^1^***  **(1.0)** | Male | 786 | Obs. | 564 | 42 | 10 | 116 | 54 | 606 | 180 |
|  |  |  | Exp. | 393 | 49 | 49 | 197 | 98 | 442 | 344 |
|  | Female | 1432 | Obs. | 964 | 220 | 102 | 146 | - | 1184 | 248 |
|  |  |  | Exp. | 716 | 179 | 179 | 358 | - | 895 | 537 |
| ***cav^1^/cav^1^***  **(4.1)** | Male | 521 | Obs. | 332 | 52 | 41 | 72 | 24 | 384 | 137 |
|  |  |  | Exp. | 260 | 32 | 32 | 130 | 65 | 293 | 228 |
|  | Female | 762 | Obs. | 494 | 76 | 94 | 98 | - | 570 | 192 |
|  |  |  | Exp. | 381 | 95 | 95 | 191 | - | 476 | 286 |
| ***moi^M12^/moi^M12^***  **(1.2)** | Male | 295 | Obs. | 174 | 35 | 32 | 47 | 7 | 209 | 86 |
|  |  |  | Exp. | 148 | 18 | 18 | 74 | 37 | 166 | 129 |
|  | Female | 122 | Obs. | 75 | 19 | 11 | 17 | - | 94 | 28 |
|  |  |  | Exp. | 61 | 15 | 15 | 31 | - | 76 | 46 |
| ***Su(var)205^4^/***  ***Su(var)205^5^***  **(4.8)** | Male | 141 | Obs. | 104 | 5 | 4 | 19 | 9 | 109 | 32 |
|  |  |  | Exp. | 70 | 9 | 9 | 35 | 18 | 79 | 62 |
|  | Female | 157 | Obs. | 89 | 31 | 10 | 27 | - | 120 | 37 |
|  |  |  | Exp. | 78 | 20 | 20 | 39 | - | 98 | 59 |
| ***tefu^atm6^/tefu^atm6^***  **(0.6)** | Male | 196 | Obs. | 158 | 12 | 2 | 16 | 8 | 170 | 26 |
|  |  |  | Exp. | 98 | 12 | 12 | 49 | 25 | 110 | 86 |
|  | Female | 464 | Obs. | 382 | 34 | 22 | 26 | - | 416 | 48 |
|  |  |  | Exp. | 232 | 58 | 58 | 116 | - | 290 | 174 |
| ***woc^rgl^/woc^rgl^***  **(2.5)** | Male | 502 | Obs. | 389 | 89 | 15 | 5 | 4 | 478 | 24 |
|  |  |  | Exp. | 251 | 31 | 31 | 126 | 63 | 282 | 220 |
|  | Female | 667 | Obs. | 522 | 121 | 18 | 6 | - | 643 | 24 |
|  |  |  | Exp. | 334 | 83 | 83 | 167 | - | 417 | 250 |
| ***nbs^1^/nbs^1^***  **(0.4)** | Male | 137 | Obs. | 104 | 15 | 2 | 10 | 6 | 119 | 18 |
|  |  |  | Exp. | 68 | 9 | 9 | 34 | 17 | 77 | 60 |
|  | Female | 285 | Obs. | 224 | 33 | 12 | 16 | - | 257 | 28 |
|  |  |  | Exp. | 142 | 36 | 36 | 71 | - | 178 | 107 |
| ***mre11^DC^/mre11^DC^***  **(0.5)** | Male | 251 | Obs. | 179 | 18 | 8 | 13 | 33 | 197 | 54 |
|  |  |  | Exp. | 125 | 16 | 16 | 63 | 31 | 141 | 110 |
|  | Female | 214 | Obs. | 150 | 43 | 11 | 10 | - | 193 | 21 |
|  |  |  | Exp. | 107 | 27 | 27 | 53 | - | 134 | 80 |
| ***eff*^Δ^*^112^/eff*^Δ^*^112^***  **(0.5)** | Male | 559 | Obs. | 431 | 33 | 19 | 25 | 51 | 464 | 95 |
|  |  |  | Exp. | 279 | 35 | 35 | 140 | 70 | 314 | 245 |
|  | Female | 1298 | Obs. | 1002 | 192 | 83 | 21 | - | 1194 | 104 |
|  |  |  | Exp. | 649 | 162 | 162 | 325 | - | 811 | 487 |
| ***peo^h^/peo^h^***  **(1.0)** | Male | 1250 | Obs. | 12 | 1 | 293 | 523 | 421 | 13 | 1237 |
|  |  |  | Exp. | 625 | 78 | 78 | 313 | 156 | 703 | 547 |
|  | Female | 906 | Obs. | 9 | 1 | 480 | 416 | - | 10 | 896 |
|  |  |  | Exp. | 453 | 113 | 113 | 227 | - | 566 | 340 |
| ***peo^h^/Df***  **(2.3)** | Male | 918 | Obs. | 159 | 1 | 168 | 290 | 300 | 160 | 758 |
|  |  |  | Exp. | 459 | 57 | 57 | 230 | 115 | 516 | 402 |
|  | Female | 1989 | Obs. | 517 | 1 | 914 | 557 | - | 518 | 1471 |
|  |  |  | Exp. | 994 | 249 | 249 | 497 | - | 1243 | 746 |
| ***peo^h^/peo^1^***  **(2.1)** | Male | 989 | Obs. | 281 | 1 | 249 | 279 | 179 | 282 | 707 |
|  |  |  | Exp. | 494 | 62 | 62 | 247 | 124 | 556 | 433 |
|  | Female | 1090 | Obs. | 237 | 1 | 485 | 367 | - | 238 | 852 |
|  |  |  | Exp. | 545 | 136 | 136 | 273 | - | 681 | 409 |
| ***peo^1^/ peo^1^; peoHA***  **(0.8)** | Male | 299 | Obs. | 94 | 9 | 33 | 81 | 82 | 103 | 196 |
|  |  |  | Exp. | 149 | 19 | 19 | 75 | 37 | 168 | 130 |
|  | Female | 624 | Obs. | 173 | 38 | 193 | 220 | - | 211 | 413 |
|  |  |  | Exp. | 312 | 78 | 78 | 156 | - | 390 | 234 |
