# Supplementary figures and images for: The Analysis of Pendolino (peo) Mutants Reveals Differences in the Fusigenic Potential among Drosophila Telomeres
Source: PLoS Genet. 2015 Jun 25;11(6):e1005260. doi: 10.1371/journal.pgen.1005260 (PMC4481407; doi:10.1371/journal.pgen.1005260)

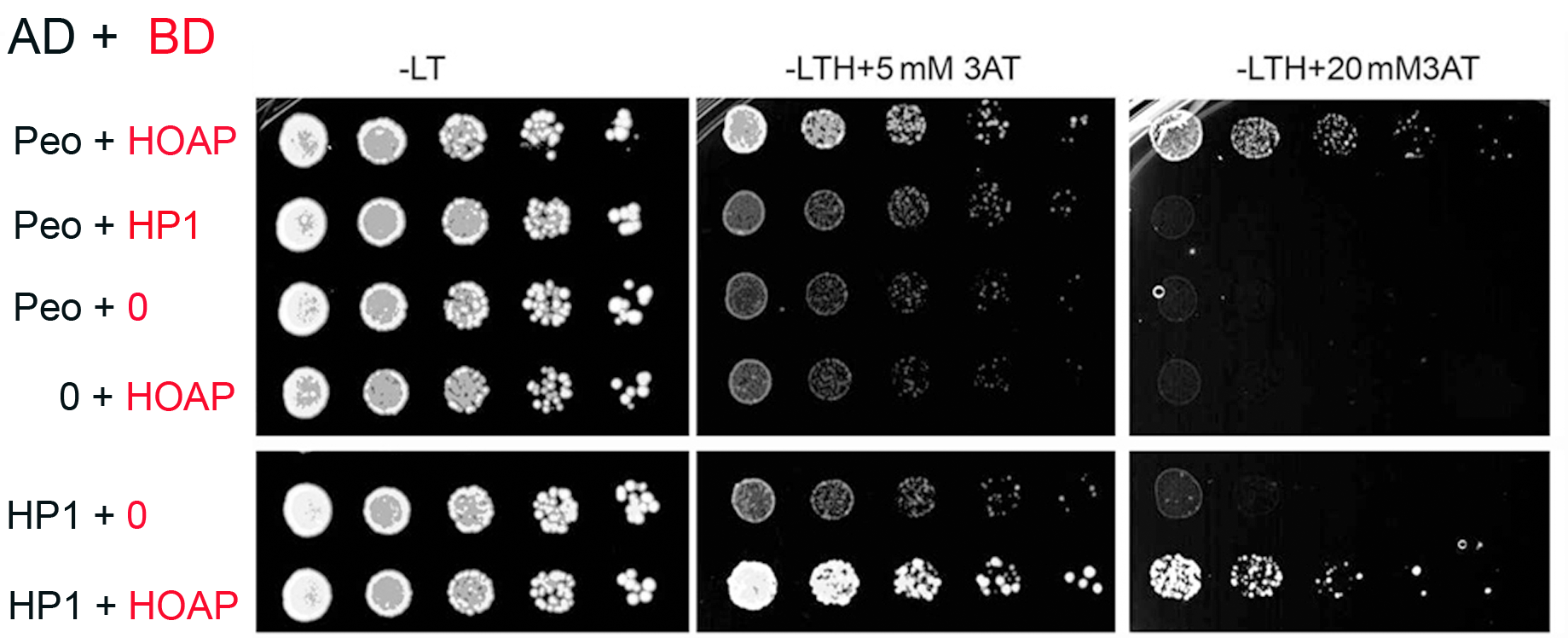

Supplement: S1 Fig — Protein interactions between Peo and HOAP or HP1a were tested by the yeast two-hybrid assay. The GAL4 DNA-binding domain (BD) and GAL4 activation domain (AD) were fused to the indicated proteins. Cells expressing the indicated combinations of bait (BD) and prey (AD) fusion proteins were plated on medium lacking Leucine and Tryptophan (-LT). The presence of physical interactions is revealed by growth on plates lacking Histidine and supplemented with 3-AT (-LTH +20 mM 3-AT). The interaction between HP1a and HOAP was used as a positive control. (TIF) [file pgen.1005260.s002.tif]

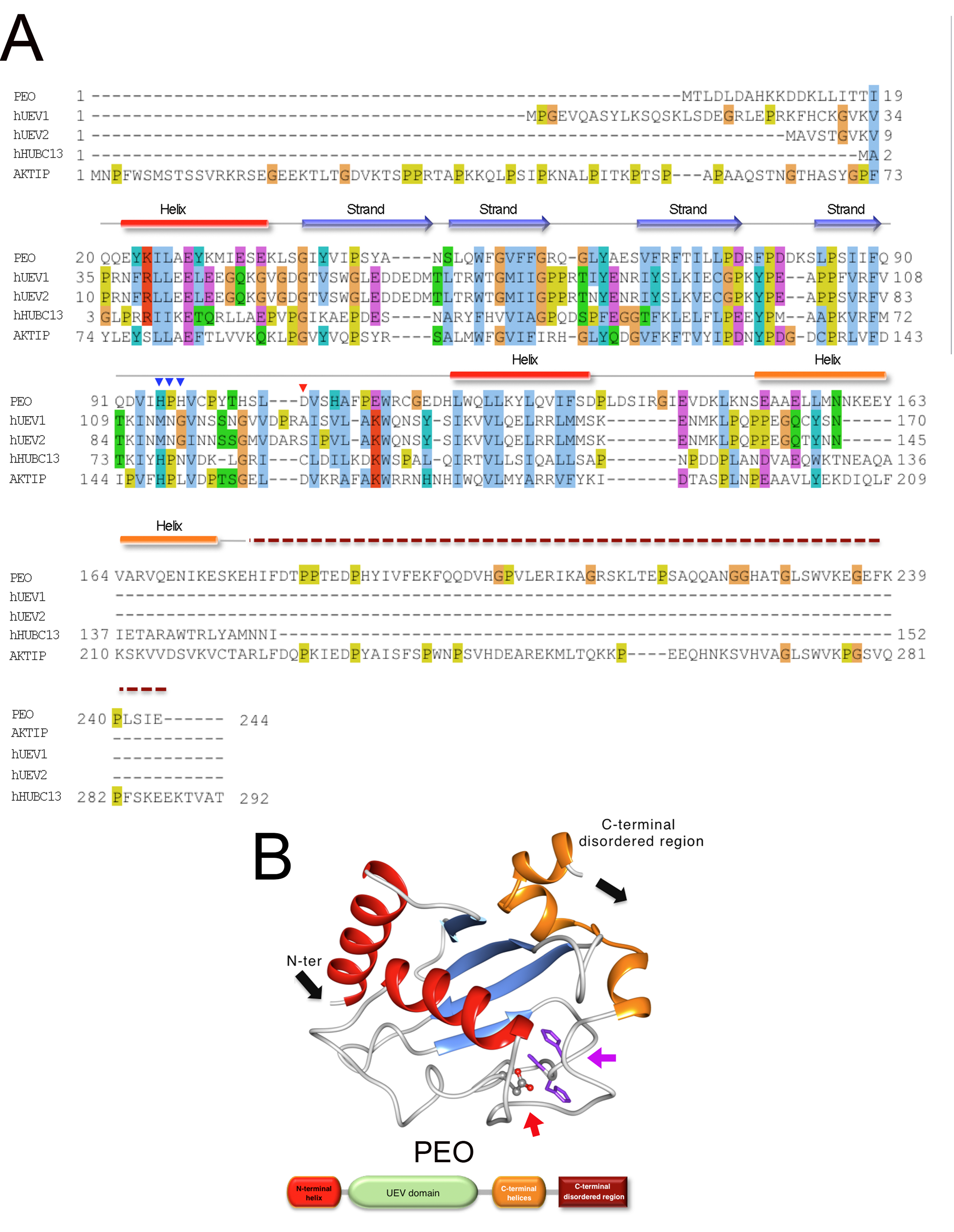

Supplement: S2 Fig — (A) Alignment of the amino acid sequence of Peo, hUEV1, hUEV2, hUBC13 and AKTIP. Secondary structure elements predicted for Peo are shown above the alignment. Red and blue arrowheads indicate the sites of the catalytic Cys (Asp in Peo) and the HPN motif (HPH in Peo), respectively. The red broken line indicates the predicted intrinsically disordered portion of Peo. (B) A three-dimensional molecular model for Peo. The arrows point to the N terminus and to the starting site of the disordered C-terminal region (not depicted); the variant Asp residue and His-Pro-His motif are represented as sticks and indicated by red and purple arrows. (TIF) [file pgen.1005260.s003.tif]

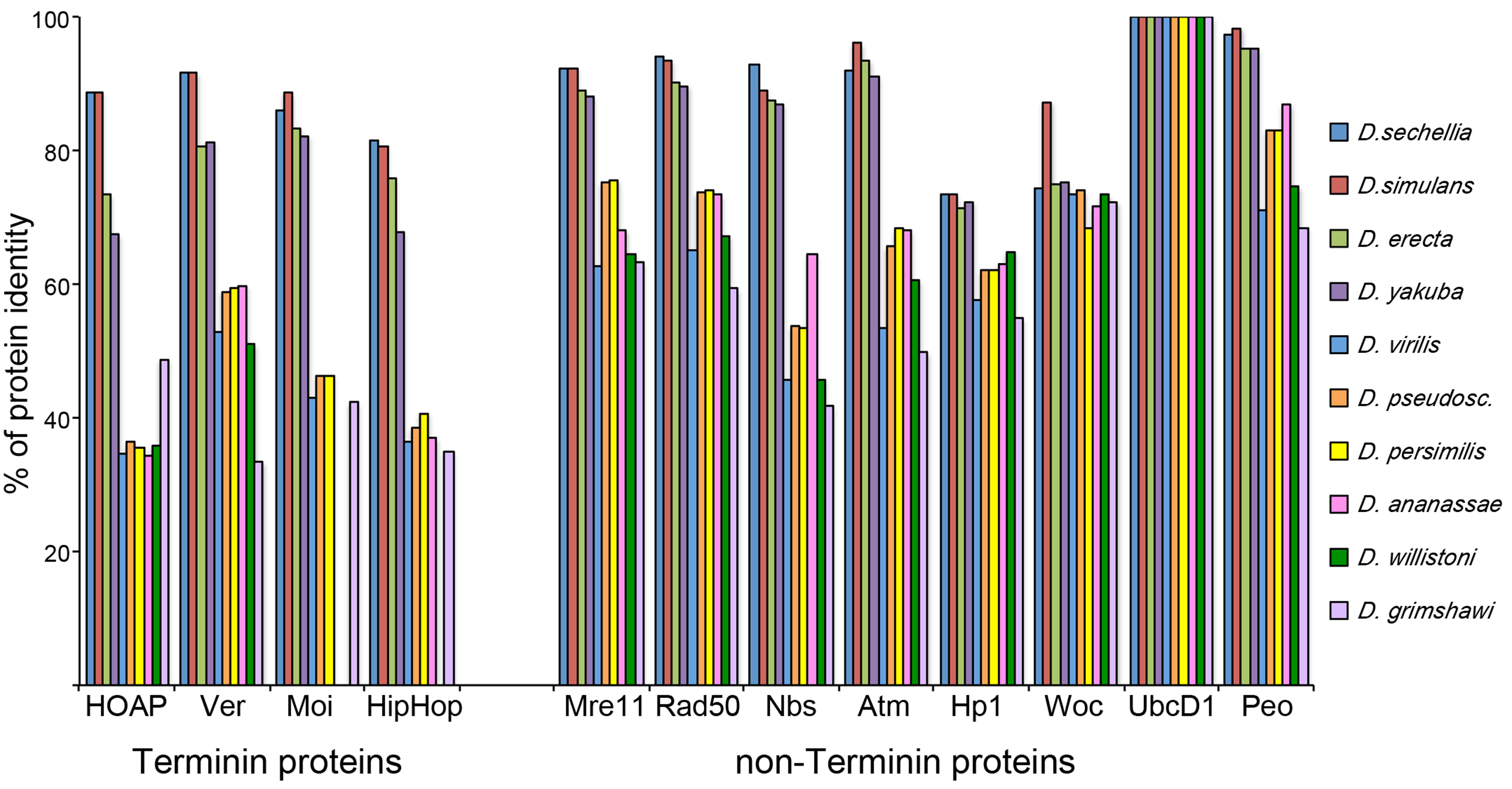

Supplement: S3 Fig — The graph shows the percentages of identity between the D. melanogaster proteins and the homologous proteins form 11 Drosophila species (D. mel, D. melanogaster; D. sim, D. simulans; D. sec, D. sechellia; D. yak, D. yakuba; D. ere, D. erecta; D. ana, D. ananassae, D. pse, D. pseudoobscura; D. per, D. persimilis; D. wil, D. willistoni; D. moj, D. mojavensis; D. vir, D. virilis; D. grim, D. grimshawi). The identity percentage is the percentage of matches between two amino acid sequences, calculated using the pairwise alignment EMBOSS Needle Software. Note that the terminin components HOAP, Moi, Ver and HipHop are poorly conserved, whereas Peo exhibits a high degree of conservation comparable to that of nonterminin proteins such as Woc or HP1a. (TIF) [file pgen.1005260.s004.tif]
